# A new method proposed to explore the feline's paw bones of contributing most to landing pattern recognition when landed under different constraints

**DOI:** 10.3389/fvets.2022.1011357

**Published:** 2022-10-10

**Authors:** Datao Xu, Huiyu Zhou, Qiaolin Zhang, Julien S. Baker, Ukadike C. Ugbolue, Zsolt Radak, Xin Ma, Fekete Gusztav, Meizi Wang, Yaodong Gu

**Affiliations:** ^1^Faculty of Sports Science, Ningbo University, Ningbo, China; ^2^Savaria Institute of Technology, Eötvös Loránd University, Szombathely, Hungary; ^3^Faculty of Engineering, University of Pannonia, Veszprem, Hungary; ^4^School of Health and Life Sciences, University of the West of Scotland, Scotland, United Kingdom; ^5^Department of Sport and Physical Education, Hong Kong Baptist University, Kowloon, Hong Kong SAR, China; ^6^Research Institute of Sport Science, University of Physical Education, Budapest, Hungary; ^7^Department of Orthopedics, Huashan Hospital, Fudan University, Shanghai, China; ^8^Faculty of Health and Safety, Óbuda University, Budapest, Hungary

**Keywords:** animal biomechanics, cat paws, feline landing, post-processing of finite element analysis, feature engineering techniques, metaheuristic optimization algorithms

## Abstract

Felines are generally acknowledged to have natural athletic ability, especially in jumping and landing. The adage “felines have nine lives” seems applicable when we consider its ability to land safely from heights. Traditional post-processing of finite element analysis (FEA) is usually based on stress distribution trend and maximum stress values, which is often related to the smoothness and morphological characteristics of the finite element model and cannot be used to comprehensively and deeply explore the mechanical mechanism of the bone. Machine learning methods that focus on feature pattern variable analysis have been gradually applied in the field of biomechanics. Therefore, this study investigated the cat forelimb biomechanical characteristics when landing from different heights using FEA and feature engineering techniques for post-processing of FEA. The results suggested that the stress distribution feature of the second, fourth metacarpal, the second, third proximal phalanx are the features that contribute most to landing pattern recognition when cats landed under different constraints. With increments in landing altitude, the variations in landing pattern differences may be a response of the cat's forelimb by adjusting the musculoskeletal structure to reduce the risk of injury with a more optimal landing strategy. The combination of feature engineering techniques can effectively identify the bone's features that contribute most to pattern recognition under different constraints, which is conducive to the grasp of the optimal feature that can reveal intrinsic properties in the field of biomechanics.

## Introduction

Much of modern human motion technology was gathered and developed from animals (1, 2). Naturalistic development of the world would not be possible without the knowledge gained from animal models (3, 4). Cats are generally acknowledged to have natural athletic ability, especially during jumping and landing (5). Cats can land safely from high positions without any injury, because of the landing buffering mechanics that they possess. The adage, “cats have nine lives”, seems applicable when we consider the animal's ability to land safely from heights (6). Several studies have reported that there have been <10% of cat's fatalities recorded while falling from heights (6–8). Vnuk et al. investigated that there was a 96.5% survival rate when a feline fell from height (6). This interesting phenomenon has attracted much research attention. Research has focused on the inner mechanical principles of the cat for providing information to reduce landing fall injuries in humans (9).

Paw pads of cats during landing are the only body parts in contact with the ground. It is believed that paw pads play an important role in the landing phase for buffering of impact force (7). The Felida family such as cats, tigers, leopards and so on are representative of the padded paw, which is commonly located beneath the distal metacarpophalangeal joints and interphalangeal joints (10). It is logical to discuss that the paw pads of cats are one of the main parts for absorbing impact force because they have relatively long tarsals and carpals. The paw pads also help to optimize stress distribution in the phalanx region (11). The paw pad is the main component area that contacts the ground in activities such as standing, jumping, walking, and running. This special morphological structure allows felines to absorb two to three times their body weight while resting on their small distal joints (7, 12). Conventional biomechanical experiments (such as animal experiments, *in vitro* cadaveric specimens, etc.) often cannot fully reflect the real biomechanical changes of internal bones, but three-dimensional finite element analysis (FEA) can simulate the complex mechanical environment in a mathematical form and provide internal mechanical information (13–15). FEA facilitates the measurement of external forces and the analysis of internal stresses during the experimental investigation, which also can provide a better understanding of the cat's special landing mechanism (1, 11).

However, the FEA also has certain drawbacks when comparing the stress characteristics of different models after the FEA (15, 16). In other words, such comparison after FEA is usually based on stress distribution trends and maximum stress values (13, 16, 17), which is a certain contingency (15, 18). For example, the maximum stress value is often related to the smoothness and morphological characteristics of the finite element model, so the comparison method of maximum stress value cannot be used to comprehensively and deeply explore the mechanical mechanism of the bone. Previous studies have explored the stress values at all nodes of a piece of bone using the *F*-test method (17). This method can effectively avoid the contingency of maximum stress value, but it ignores the effective information of stress distribution characteristics. Therefore, it has become a challenge in the field of biomechanics in the post-processing of FEA to analyze the stress distribution characteristics of bones effectively while avoiding the chance of the existence of stress extremes (15, 19–21).

In recent years, machine learning methods that focus on feature pattern variable analysis have been gradually applied in the field of biomechanics (1, 22–24). Meanwhile, the progress of motion capture technology, mechanical sensing technology, and signal processing technology makes biomechanical data acquisition diversified and refined, which provides the prerequisite for the application of big data-driven machine learning methods in the feature recognition and selection in the field of biomechanics (1, 24, 25). Metaheuristic optimization algorithms are a fascinating research hotspot in the field of machine learning, and it has been significant in solving complex and difficult feature optimization problems (26, 27). At present, there have been a large number of studies using metaheuristic optimization algorithms to select and classify characteristics of biological data (28, 29). Particle swarm optimization (PSO) is a classical and widely researched algorithm in the field of metaheuristics, which aims to deal with optimization problems in continuous or discrete spaces based on population search (30, 31). The construction of a bone stress distribution pattern recognition and feature selection model based on PSO can provide some methodological reference for the problem of stress feature exploration in the field of biomechanics and provide unique new insights into the results.

Therefore, this work aimed to explore the cat forelimb paw biomechanical characteristics when landing from different heights by using FEA, and feature engineering techniques for post-processing of FEA. Specifically, the ground reaction force (GRF) data waveform during the cat landing was first reconstructed using principal component analysis (PCA), and the optimized data was substituted into a finite element model simulation to calculate the bone stress distribution. After that, by extracting the node stress values of each bone in the finite element model as model input data, a feature selection model was constructed based on PSO in the metaheuristic optimization algorithm to select the optimal bone stress distribution features that can identify landing patterns when landing from a different height. Meanwhile, a feature classification and recognition algorithm model was constructed to determine the accuracy of recognizability of each bone stress distribution feature for landing patterns when landing from a different height. Finally, the aim of exploring the cat landing patterns characteristics and law during landing from different heights was achieved by combining the above results, and the advantages of FEA post-processing based on feature engineering techniques have also been demonstrated.

## Materials and methods

### Animals

**Figure 1:**
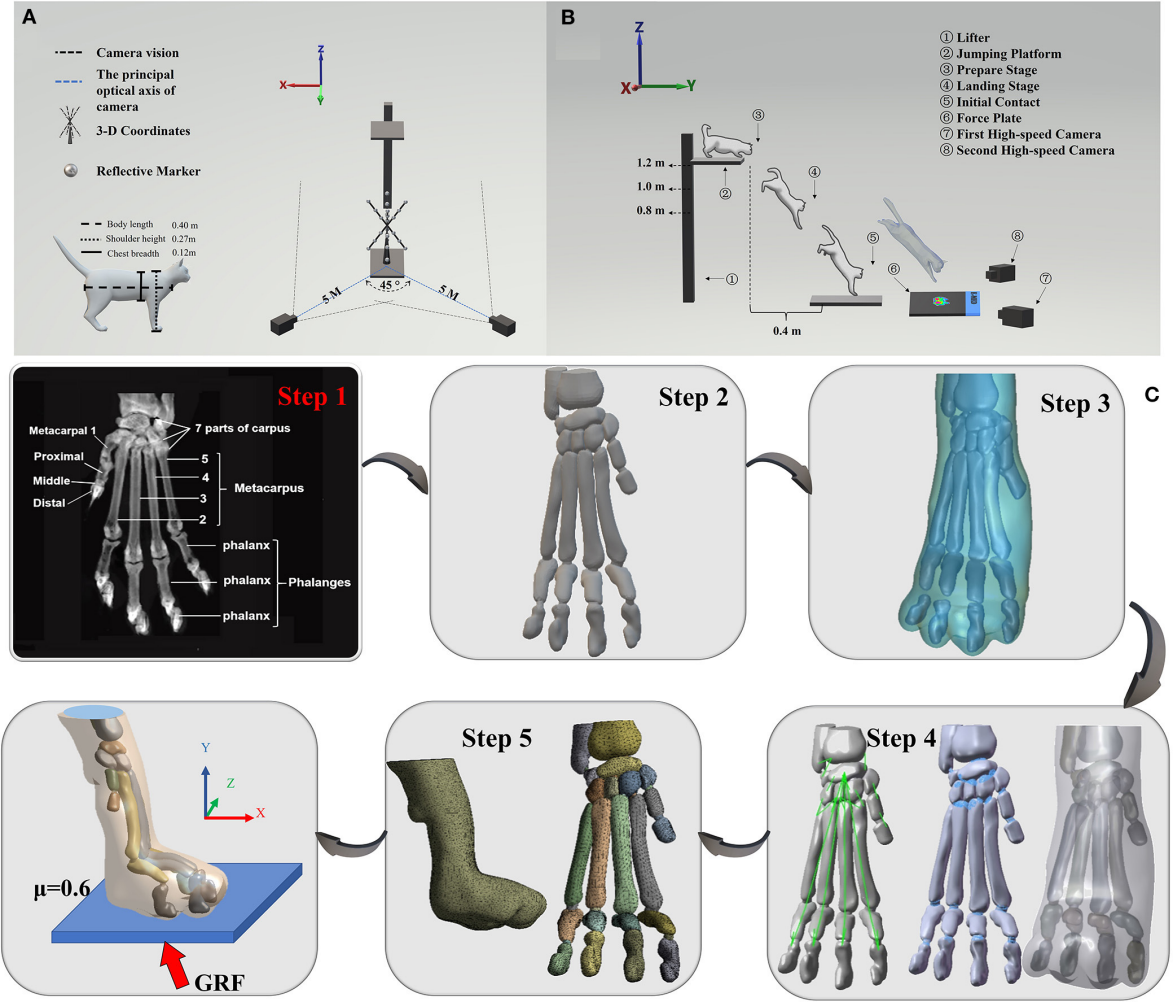
**(A)** Illustration of the position of 3-D coordinates and two high-speed cameras. **(B)** Illustration of cat landing experimental procedure from the ready position of jumping platform to initial forelimbs contacting the force plate. **(C)** Illustration of the process of FEA. Step 1 is to obtain the coronal CT images of the right forelimb paw. Step 2 is the 3D model obtained by processing CT images through Mimics software, and then importing the 3D model into Geomagic software (Step 3) for post-processing such as noise reduction, spike removal and smoothing. After that, Step 4 was performed by using SolidWorks software to get cartilage, ligaments, and soft tissue (from left to right). Finally, Step 5 was executed by using ANSYS Workbench software to grid processing, then load and boundary conditions were applied to execute FEA.

Written agreement from the breeder in the local area was obtained for the voluntary involvement of a healthy male domesticated cat: aged 2.85 years, body mass of 4.32 kg (Figure 1A shows the specific body length of the test cat). There was a comprehensive clinical assessment prior to data collection in order to guarantee that there were no health conditions that would affect the study's results. A computerized tomography (CT) scan of the cat was taken. The CT scan was conducted by a veterinarian at a pet hospital. In order to ensure that there were no health issues or foot injuries, the cat was inspected by a veterinarian. The Animal Care and Use Ethics Committee of Ningbo University gave its approval to this research (NBUAEC20200621).

### Experiment protocol and procedures

All tests were performed in the biomechanics laboratory at Ningbo University Research Academy of Grand Health. The landing task was performed on a force platform (Kistler, Switzerland) using a 1,000 Hz sampling frequency for GRF data collecting. During each landing task, kinematic data were collected using two high-speed cameras (Fastcam SA3, Photron, Japan) set at 1,000 Hz. At the same time, the other landing task was performed on a pressure sensing mat EMED-AT system (Novel, Germany) to collect the E-med data (EMED-AT system: 700 × 403 × 15.5 mm with a sensor area of 475 × 320 mm, containing 6,080 sensors with a recording frequency of 100 Hz). The cat was completely acclimated to the setting (test room) prior to data collection, with toys and food used to entice the cat's interest. To ensure a smooth experiment, the cat was brought to the laboratory by its owner before the official start of the trial. This procedure was repeated three times a week for 1 h each time until the cat could be lured by food and toys and precisely leap to the appropriate place (the force platform). Three heights of 0.8, 1, and 1.2 m were taken as the heights selected for this experiment. Twenty groups of data were collected by a force platform and E-med for each height, and a total of 120 groups of data were collected.

The cat owner urged him to sit in a squat posture on the leaping platform while the table height was changed to the exact height necessary. To minimize erroneous data collection due to fatigue, a 5-min break was implemented between each landing task. The cat's head and body were both facing forward when it fell, so there was no obvious tilt to the body. When the cat's forelimb landed in the defined region and the cat proceeded to travel ahead from the indicated area, the experiment has judged a success. There were no injuries or negative responses following the experiment. Two high-speed cameras were mounted at the diagonal level of the force plate at a distance of 5 m from the landing target region, producing a 45-degree angle between the major optical axes of the two cameras, as shown in Figure 1A. Three-dimensional (3-D) coordinates were put in the center of the force platform to create the space coordinate. Figure 1B shows the landing test procedure of the cat from a preparation stage to an initial contact phase.

### Data processing and statistical analysis

The first point of contact with the force plate was determined using a vertical GRF > 10 N (1, 32). The landing phase was defined as the first point of contact (0% landing phase) to maximum elbow flexion from the first peak vertical GRF time point to the second (100% landing phase). The GRF data was filtered using Butterworth lowpass filters (filter order: fourth-order zero-phase lag, cut-off frequency: 50 Hz) (33). SIMI-Motion 7.50 is a motion simulator developed by SIMI-Motion (Simi Reality Motion Systems GmbH, Munich, Germany), which was used to analyze the cat landing phase. After that, the elbow sagittal plane joint angles were taken as an output from SIMI -Motion, and the time point corresponding to the maximum elbow flexion angle was derived to intercept the data waveform of the GRF. Then, each landing height (0.8, 1.0, and 1.2 m) of each direction (X-axis: lateral and medial GRF; Y-axis: anterior and posterior GRF; Z-axis: vertical GRF) of the determined GRF data were expanded into 101 data points using a self-written MATLAB script, which represents the 0–100% landing phase (1). Finally, the data waveform of the GRF was run in MATLAB by a custom MATLAB script to execute the PCA to reconstruct the waveforms of the principal GRF.

At the same time, the time point corresponding to the maximum elbow flexion angle was also used to determine the E-med data. The SPSS 24.0 for Windows™ software was used for statistical analysis (SPSSs Inc., Chicago, IL, USA). Prior to statistical analysis, the Shapiro Wilk normality test was applied to all E-med data. If non-conformity was observed then the Wilcoxon matched-pairs signed-rank test was conducted for non-parametric data. Independent *t*-tests were performed to determine if there were any significant differences in different biomechanics values between left and right forelimbs. A one-factor repeated ANOVA was performed to determine the effect of landing heights during the landing phase on the right forelimb. The Least Significant Difference (LSD) was used in the post-test of analysis of variance, and the *P*-value was also corrected based on the result of the post-test.

### Principal component analysis reconstructed data waveform of ground reaction force

PCA is a multivariate statistical analysis technique that uses orthogonal rotation transformation to convert multiple indexes into several comprehensive indexes to reduce dimensionality and sacrifice as little information as possible (34). The principle component is the name given to the comprehensive index produced by transformation, in which each principal component is a linear combination of the original variable and is unrelated to the others (1, 34, 35). When investigating complex problems, it is possible to consider only a few principal components without missing too much information. As a result, it is simpler to identify the major contradiction, disclose the regularity between the internal variables of objects, and reduce the problem in order to increase analytical efficiency. See Supplementary Text 2 for more details on the application of PCA in current research.

### Finite element analysis technology simulated the bone stress distribution of cat claw

The specific FE model feline paw model was created using Computer Tomography (CT) images. CT scans were obtained and conducted at a pet hospital by a qualified veterinarian. Before obtaining the CT data, the cat was examined by a veterinarian to confirm that there were no health problems or foot injuries.

The whole process of FEA is shown in Figure 1C. Coronal CT images of the whole body were collected with a space interval of 0.5 mm in the unloaded position, while only the right forelimb paw was analyzed in this experiment (1, 15). The body of the cat was oriented in the scanner in a specific way to mimic the posture of the cat landing. The structures of 23 bones, which included 1 radius, 1 ulna, 7 carpus, 5 metatarsals, and 9 components of the phalanges together with the encapsulated volume were segmented using MIMICS 20.0 (Materialise, Leuven, Belgium). To obtain the boundaries of the skeleton, the bones were saved in STL format. Secondly, they were imported into specific software (Geomagic, Inc., Research Triangle Park, NC, United States) for post-processing. This included noise reduction, spike removal, and smoothing. The file was then imported to SolidWorks (SolidWorks Corporation, Massachusetts, 2017) in Iges format SolidWorks. SolidWorks was utilized for the conversion of all volumes to solid parts individually. To simulate the real situation of the cat's paw, the solid volume of the articular cartilaginous structure was shaped. Eventually, 23 cartilages were created according to the feline paw anatomical structure. Additionally, the encapsulated soft tissue was built by subtracting all bones and cartilages and converting them into a solid format. The ligaments were then generated based on anatomical characteristics (36). All 76 parts of the paw, which included 23 bones, 23 cartilages, 30 ligaments, and an encapsulated soft tissue. Using ANSYS Workbench 17.0 (ANSYS, Inc., Canonsburg, United States) for meshing each part. The solid model of each bone was divided into a high-quality mesh using the self-adapting dynamic biomechanical FE grid of the Modeler. The length of the mesh was designated as 1–2 mm. Finally, load and boundary conditions are applied, and FEA is performed on the model. More details about the material properties, loading, boundary conditions and connections for FE models are shown in Supplementary Text 2.

### Optimal feature selection of landing patterns based on bone stress distribution

Based on the three landing heights, the optimal features can be selected in two cases: landing from 0.8 m vs. landing from 1.0 m, landing from 1.0 m vs. landing from 1.2 m. Data was entered 5 times in each of the two comparisons, a total of 10 data sets: *M*_*data*1_, *M*_*data*2_, *M*_*data*3_, *M*_*data*4_, *M*_*data*5_, *M*_*data*6_, *M*_*data*7_, *M*_*data*8_, *M*_*data*9_, *M*_*data*10_. Refer to Supplementary Text 2 for details of what each dataset represents.

When the metaheuristic swarm intelligence algorithm performs optimization calculations, the population of individuals represents different meanings for different optimization problems (26, 37). The essence of feature selection in this study is binary optimization. Specifically, the present work uses the construction of a binary particle swarm optimization feature selection algorithm model to select the stress characteristics of the cat's metacarpal and the phalanx of claws that can identify the landing patterns of cats at different altitudes. At each time step, the PSO idea involves accelerating each particle toward its *Pbest* and *Gbest* positions by modifying its velocity (global version of PSO). Random numbers are created for *Pbest* and *Gbest* acceleration sites, which are weighted by a random term. For the binary particle swarm optimization (BPSO), the cognitive factor was set to 2, the social factor was set to 2, and the inertia weight was set to 0.9. The population in BPSO is referred to as a swarm, which consists of *N* particles that move around the search space in multiple dimensions. Potential solutions are represented by particles that travel across the search area to find the best option. According to its own experience and knowledge, each particle looks for the global maximum or minimum. More detailed descriptions of BPSO are shown in Supplementary Text 2.

After optimization, the representation of the feature selection result is limited to 0 and 1. The value 0 means that the feature is not selected, and the value 1 means that the feature is selected (37, 38). When optimizing the selection of features, the individual solution of the swarm can be regarded as a one-dimensional vector, and the original data value of each dimension is compared to 0.5. If the value is ≥0.5, the value is defined as 1, and the feature is selected; otherwise, the value is defined as 0, and the feature is unselected. For example, if the solution *X* = *a*{0.82, 0.63, 0.35, 0, 0, 1, 0.98, 0.87, 0.14}, it represents six features (1st, 2nd, 6th, 7th, 8th) are selected. The number of iterations for all optimization algorithms was set as 100, and the fitness function can be defined as:


(1) $$\begin{array}{l} Fitness\;value=\;\alpha E_{R}+\left(1-\alpha \right)\frac{\left|R\right|}{\left|S\right|} \end{array}$$

(2) $$\begin{array}{l} E_{R}=\frac{Number\;of\;wrongly\;predicted\;instances}{Total\;number\;of\;instance} \end{array}$$

where the *E*_*R*_ is the error rate calculated by the learning algorithm, |*R*| is the feature subset's length, |*S*| denotes the total number of features, α is the parameter of control of the weight (between the ratio of selected features and error rate). In this study, the α was set to 0.9 since the classification performance was the most essential measurement. The k-nearest neighbor (KNN) algorithm was selected as the learning algorithm for fitness evaluation (Supplementary Text 2). For performance evaluation, the hold-out validation method was applied, and the value ratio of validation data was set as 0.2.

After that, the top three features that have been selected the most times based on 20 random seeds were selected as the final extracted features. The realization of the whole algorithm is through MATLAB self-written scripts based on previous research and MATLAB built-in toolbox.

### Feature classification and recognition based on bone stress distribution

For the classification and recognition algorithm model of landing features, the current research is also divided into two cases to test the classification and recognition accuracy of features. A total of 10 data sets (*M*_*data*1_ to *M*_*data*10_) were substituted into the constructed model. In this study, the KNN (39), support vector machine (SVM), and artificial neural network (ANN) (40) were selected to classify in this study because they have been widely used in pattern recognition and classification. More detailed descriptions of recognition and classification model building are shown in Supplementary Text 2. The 10-fold cross-validation was used in all classification models.

## Results

### Pressure and force distribution on cat from E-Med

**Figure 2:**
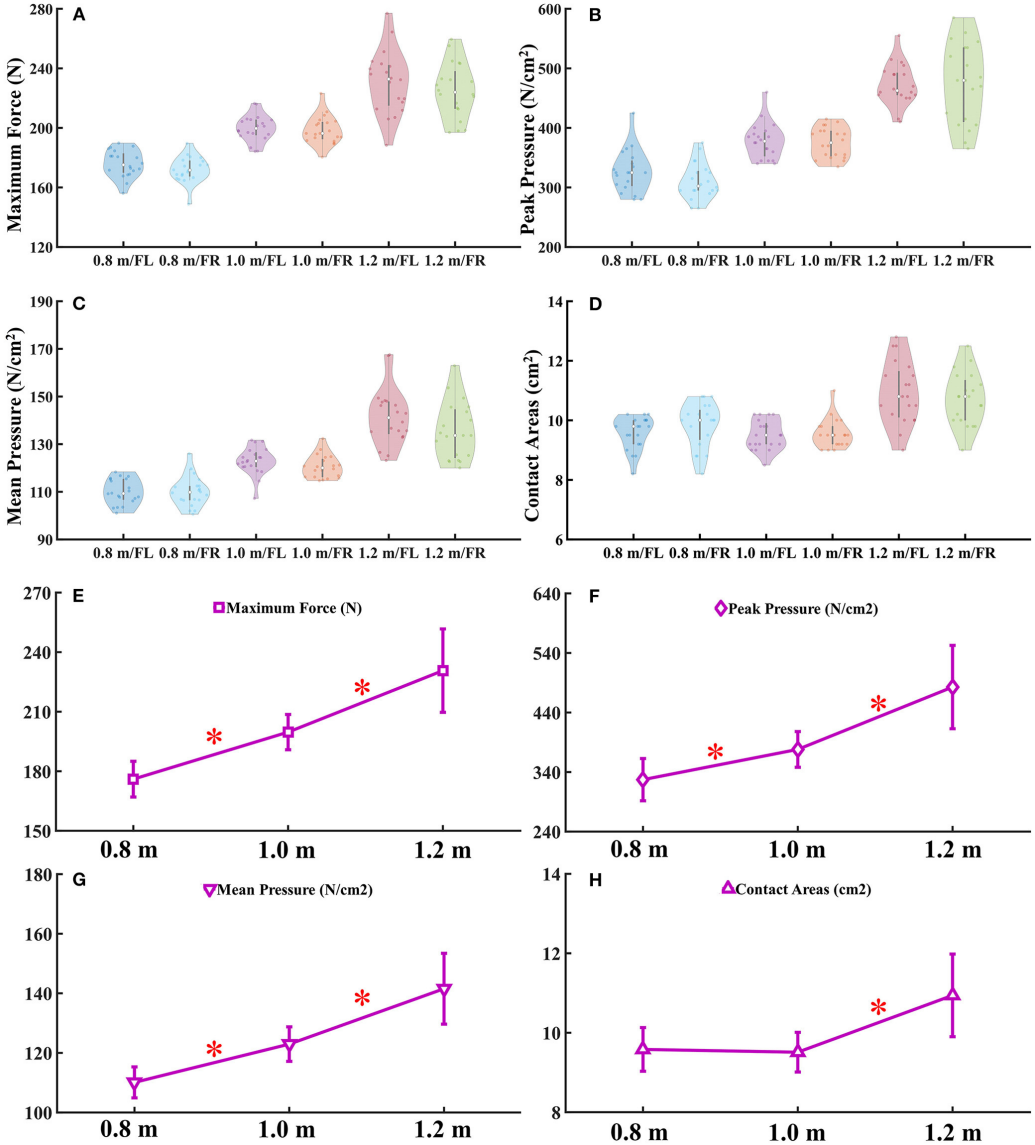
**(A–D)** The violin plot of E-Med data distribution. **(E–H)** Comparison of forelimb paw right in maximum force, peak and mean pressure and contact areas during landing from different heights. FL, Forelimb paw left; FR, Forelimb paw right; “*” represents significant difference between heights. 1 MPa = 1,000 N/cm^2^.

There were no significant differences found in maximum force (Figure 2A), peak pressure (Figure 2B), mean pressure (Figure 2C), and contact areas (Figure 2D) during the landing task from three different heights between left and right forelimb paw. The detailed data analysis results are shown in Supplementary Table S3. Significant differences were found in right forelimb paw at maximum force (Figure 2E), peak pressure (Figure 2F), mean pressure (Figure 2G), and contact areas (Figure 2H) during the landing task between 0.8, 1.0, and 1.2 m, respectively. The detailed data analysis results are shown in Supplementary Table S4.

### Reconstructed waveforms of principal component analysis

**Figure 3:**
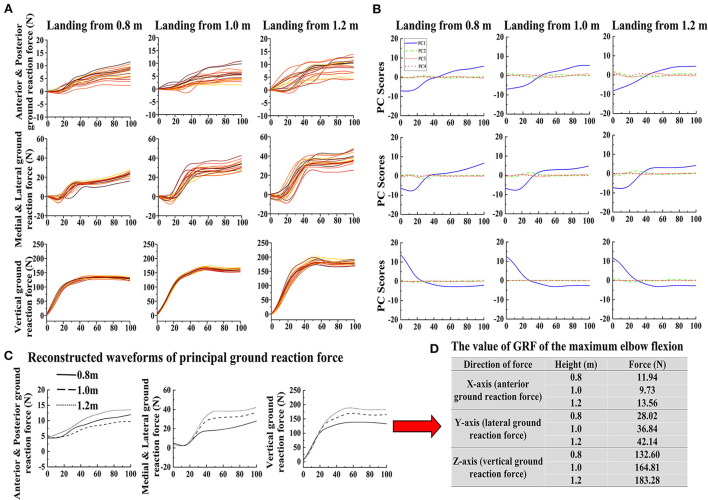
**(A)** The raw data waveform of GRF for each direction (X-axis: anterior and posterior GRF; Y-axis: lateral and medial GRF; Z-axis: vertical GRF) in each height (0.8, 1.0, and 1.2 m). **(B)** The first four PC scores *PC*_*i*_ of each landing height and each direction GRF. **(C)** The reconstructed waveforms of each landing height and each direction GRF. **(D)** The extracted principal GRF value of the maximum elbow flexion data point based on the reconstructed waveform in each direction during landing from different heights.

The data waveform of GRFs in three directions when the cat landed from three heights are shown in Figure 3A, with a total of 20 waveforms for each case. Results of PCA based on these data are reported. The first four PC scores *PC*_*i*_ of each landing height and each direction GRF are shown in Figure 3B. For the first PC scores PC_1_ of GRF, the PC_1_ covers the most important characteristic information of the waveform. This highlighted that PC_1_ can reconstruct the principal GRF $\vec{PF}$ (1, 35). Therefore, the first PC scores PC_1_ was selected to reconstruct the principal GRF $\overset{}{PF}$ in each direction. The waveform difference between different landing height of reconstructed principal GRF $\overset{}{PF}$ is shown in Figure 3C. According to the reconstructed waveform of principal GRF, the GRF value for each landing height of the maximum elbow flexion was extracted (the detailed values are shown in Figure 3D). Finally, the GRF data values of each landing height at the time point of the end of the landing phase (maximum elbow flexion) are extracted from the reconstructed waveform and substituted into the finite element model to investigate the stress distribution of the cat right forelimb paw bone (metacarpal and phalanx).

### Finite element model validation

During the process, 4-note linear tetrahedral elements were used on the irregular geometries such as bones, cartilage, and encapsulated tissue. The established three-dimensional FE models include 215,885 elements, 359,299 nodes. The structures of 23 bones included 1 radius, 1 ulna, 7 carpus, 5 metacarpals, and 9 components of the phalanges. Referring to the numerical model of the human foot, the FE model foot models were validated by plantar pressure distribution (1). Detailed procedures and results are provided in Supplementary Text 1. The results showed that the numerically determined pressure distribution in the left forelimb paw was in good agreement with experimental data (Supplementary Figure S1).

### Right forelimb paw stress distribution

**Figure 4:**
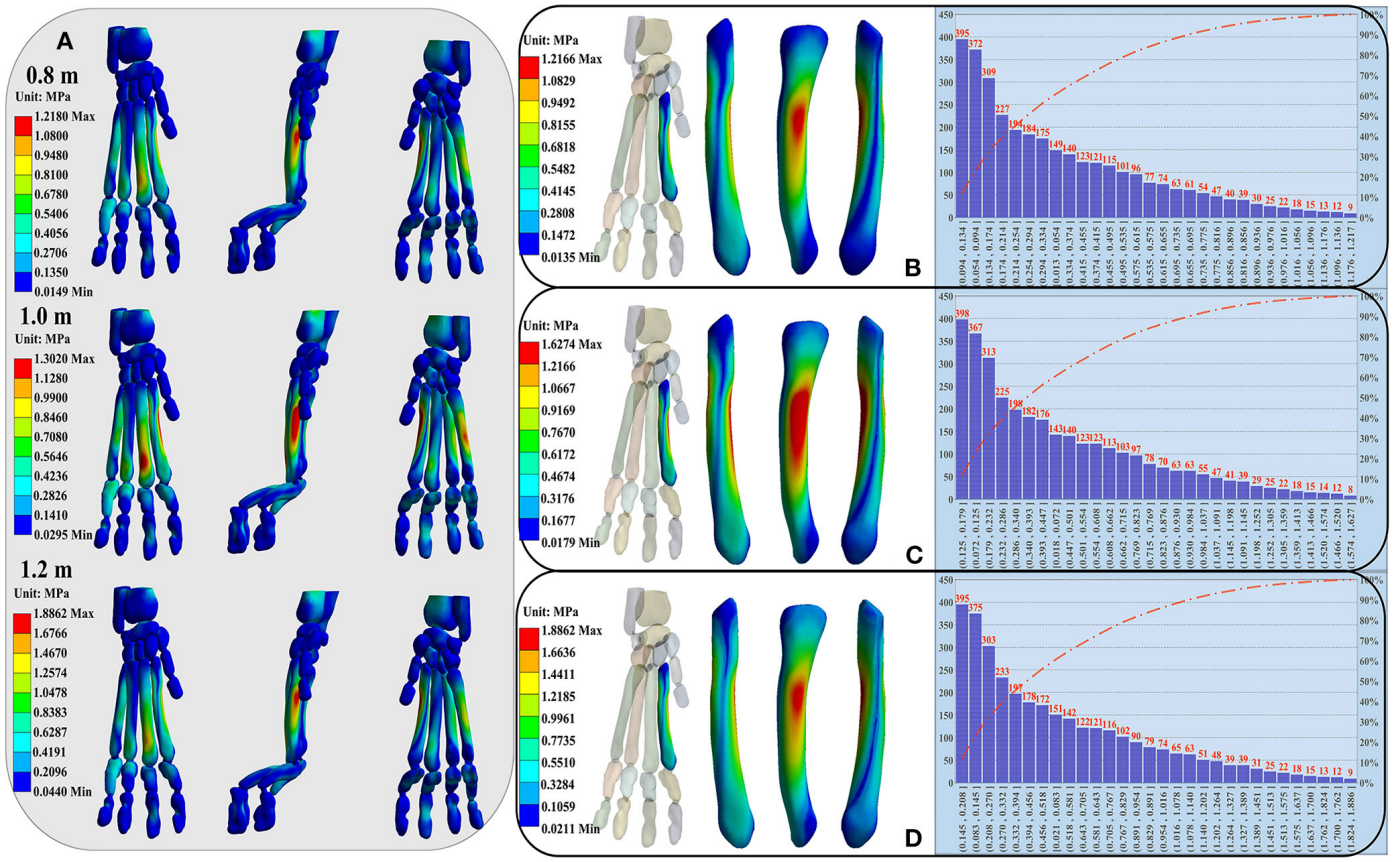
**(A)** Illustration of the right forelimb paw stress distribution of the cat landing from different heights. From left to right are front, side and back views of the overall stress distribution. **(B–D)** Display the detailed stress distribution heatmap (left side) and Pareto distribution (right side) results of stress values at all nodes of MP2. From left to right are front view, side view and back view of the MP2. The stress distribution from top to bottom corresponds to the landing heights of 0.8, 1.0, and 1.2 m.

Twelve bones surrounding the cat's paw pad were selected as features. The number of finite element model nodes corresponding to each bone is shown in Supplementary Table S5. The overall stress distribution of the right forelimb paw is shown in Figure 4A. The stress is mainly concentrated at the metacarpal and proximal phalanx. The MP2 had the highest stress level. Specifically, the stress was mainly concentrated in the middle and rear of the MP2 and the MP5, and the distal end of the MP3. The pressure between the MP4 and the MP5 was similar, but the stress distribution of the MP4 was more uniform. The maximum stress value of the MP5 was always greater than that of the MP4 during landing from each height. The proximal and distal phalanges were less stressed. The detailed stress distribution heatmap and Pareto distribution results of stress values at all nodes of MP2 are shown in Figures 4B–D. The detailed stress distribution of other 11 bones are shown in Supplementary Figures S2–S12.

The left side of the figure is the detail heatmap diagram of stress distribution, and the right side is the distribution diagram of stress values of all nodes [Pareto distribution (41): the stress values of all nodes are arranged in descending order, and then divided into 30 distribution ranges in order]. The number of nodes were arranged according to the stress value from top to bottom, each bones stress distribution ranges of all nodes and of the last 50, 80, 90, and 95% nodes, as well as the first 5% nodes are shown in Supplementary Table S6. For the MP2, the stress was mainly concentrated in the middle and rear. The stress distribution ranges of all nodes were 0.0135–1.2166, 0.0179–1.6274, and 0.0211–1.8862, respectively, the stress distribution ranges of the last 50% nodes were 0.0135–0.2550, 0.0179–0.3418, and 0.0211–0.3936, respectively, the stress distribution ranges of the first 5% nodes were 0.8772–1.2166, 1.1748–1.6274, and 1.3588–1.8862, respectively (Supplementary Table S6; Figures 4B–D).

### Feature selection results based on the bone stress distribution

**Figure 5:**
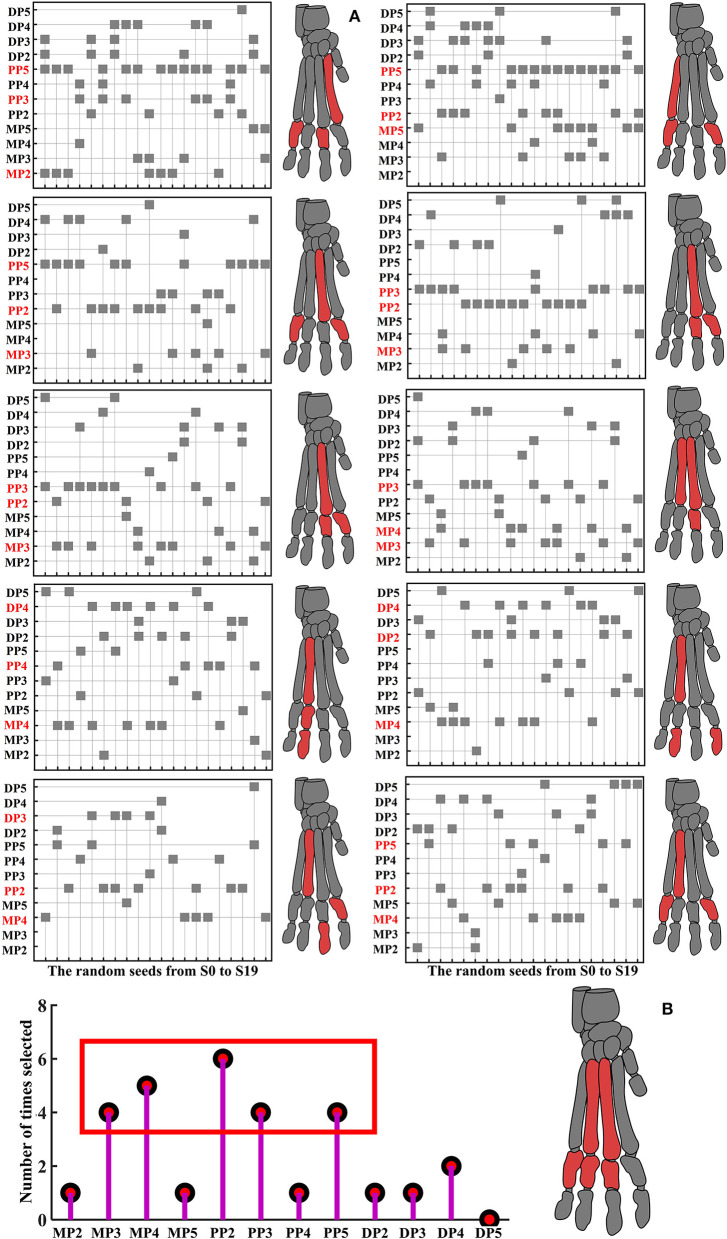
Detailed results of features selected in each contrasting situations in each random seed. **(A)** The left side is the feature selection results based on the data of landing from 0.8 to 1.0 m, and the right side is the feature selection results based on the data of landing from 1.0 to 1.2 m. From top to bottom are the results based on the data of stress value corresponding to all nodes, the data of the first 2,000 nodes with the highest stress values, the data of the first 1,000 nodes with the highest stress values, the data of the first 500 nodes with the highest stress values, the data of the first 200 nodes with the highest stress values, respectively. The shaded square indicates that this feature was selected under the random seed, the claw bones marked red are the top three stress distribution features selected the most times. **(B)** The number of times of each selected feature based on the three stress distribution features selected the most times for each contrasting situations.

For each random seed (20 random seeds) in each contrasting situations (10 kinds of input data), the results of fitness value are shown in Supplementary Figure S13. The detailed results of the feature selected in each contrasting situations in each random seed based on the constructed feature selection algorithm model are shown in Figure 5A. See Supplementary Text 1 for the detailed description of Figure 5A. Finally, the stress distribution features that contribute most to the landing pattern recognition at different heights mainly focused on MP3, MP4, PP2, PP3, PP5 (Figure 5B). In terms of MP4 and PP2, PP2 basically exists in most node control cases, and MP4 mainly exists in the results based on the stress value data corresponding to the first 200 and 500 nodes with the highest stress values.

### Feature classification and recognition results based on the bone stress distribution

**Figure 6:**
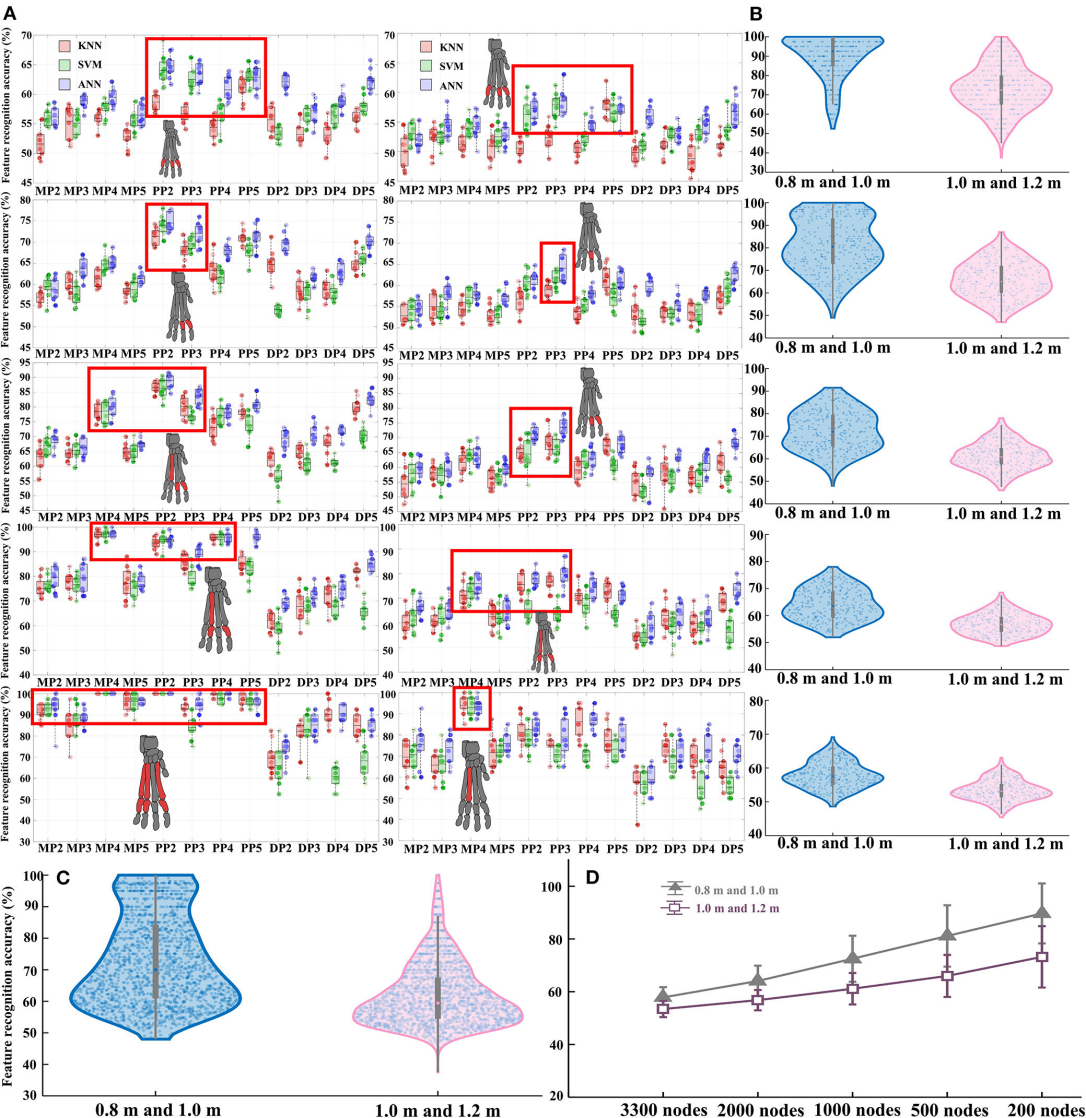
**(A)** Detailed results of features classification and recognition accuracy rate in each contrasting situation of the three different classification algorithm models. The left side is the feature classification and recognition results based on the data of landing from 0.8 to 1.0 m, and the right side is the feature classification and recognition results based on the data of landing from 1.0 to 1.2 m. From top to bottom are the results based on the stress value data corresponding to all nodes, the data of the first 2,000 nodes with the highest stress values, the data of the first 1,000 nodes with the highest stress values, the data of the first 500 nodes with the highest stress values, and the data of the first 200 nodes with the highest stress values, respectively. In the red box are the features with higher classification and recognition accuracy than other features, corresponding to the claw bones marked in red. **(B)** Total classification and recognition accuracy data distribution of all features in each node control case. **(C)** Total classification and recognition accuracy of all features under two contrasting situations. **(D)** The final classification recognition accuracy trend of all features in each node control case.

The detailed results of features classification and recognition accuracy rate in each contrasting situation of the three different classification algorithm models are shown in Figure 6. In each contrasting situation, the total exact classification recognition accuracy obtained by the three classification models is shown in Supplementary Table S7. The classification of each bone stress distribution when landing from different heights can be objectively and accurately detected by combining the three classification models. The bone stress feature recognizability between landing from 0.8 m and landing from 1.0 m is significantly higher than that of landing from 1.0 m and landing from 1.2 m (Figures 6B,C). For the results based on the data of stress value corresponding to all nodes, they both show a poor classification in the landing height between the 0.8 and 1.0 m, 1.0 and 1.2 m. As the stress value of the selected nodes gradually increases (from the data of stress value corresponding to all nodes to the data of the first 200 nodes with the highest stress values), the classification and recognition accuracy of the stress distribution features of each bone gradually increases (Figure 6D; Supplementary Table S7). In each contrasting situation, features with higher classification recognition accuracy compared with other features are shown in the red mark in Figure 6A, and these features contribute more to the recognition of different landing patterns. More details on this are shown in Supplementary Text 1.

## Discussion

The current work investigated the biomechanical characteristics of the cat forelimb paw during landing from different heights using FEA and feature engineering techniques for post-processing of FEA results. The main contribution of the current study is to fill the field gap of cat's paw biomechanics during landing, and the proposed combination of feature engineering technology to solve the problems that include incomplete analysis, and difficulty in feature mining in the post-processing of FEA.

This study explored the differences between the left and right forelimbs for maximum force, peak and mean pressure and contact areas during a landing task from different heights, and the results show that there were no significant differences between left and right forelimbs. A previous study also demonstrated that when a cat jumps from a height of 1 m, the force on its forelimbs is equal (42). It was consistent with our results. Wang et al. proved that when a cat jumps from a height of 30 and 50 cm, the force on its forelimbs is equal (43). This work further demonstrated that the contact area of paw pads is equal. Slingerland et al. concluded that cats are forelimb-dominant, and it might explain the findings of this study (44). The forelimbs are the dominant part of the cat, so in the landing process, the forelimbs also play the role of direction and posture control. When falling from a high place, due to this excellent symmetry, the impact force generated by the landing can be fully and evenly distributed to the two forelimbs, so that forces can be transmitted to other joints in a positive and even manner. This explains in part and is one of the reasons why cats can fall from great heights without injury. However, Wang et al. also mentioned that the symmetry of the forelimbs would increase with increases in jumping heights (11). This was inconsistent with our results, we did not find any asymmetry values at 0.8, 1.0, and 1.2 m, which is probably because these heights are still within the acceptable range for a cat to land normally.

In a previous study, the findings indicated that the muscle or soft tissue of cats absorbed impact force when landing or walking (40). We partly agree with the findings of the T. Kohonen et al. According to our study, the skeleton of the cat also plays an important role when cats perform landing or walking tasks. It is difficult to confirm which parts are more important and contribute the most. Xu et al. believe that the MP pad has a larger surface area than the digital pad, and its special columnar structure provides better support for the body while playing a dominant role in distributing and absorption of impact (1, 45). From the view of the overall stress distribution of the right forelimb paw, the stress is mainly concentrated on the rear of MP2, MP5 and the front of MP3, MP4, and the average stress of MP except MP2 tends to be consistent. The uniform stress distribution during the landing of the cat reduces the risk of fracture, which may be related to the small range of motion of the distal joint. Previous studies have shown that there was a limit on the wrists during cat movements (12). The carpal bone, which is part of the wrist joint, shows more degrees of freedom than the hinged joint (36). In order to counteract the multi-dimensional motion of the wrist, the movement of the MP joint may also be limited to maintain the stability of the upright posture of the lower extremities. The cat's forelimb is subjected to a greater impact during landing, and the forelimbs of the cat would move toward the palm (46). Under these conditions, the wrist provides full engagement in its supporting function, while increasing the contact area between the ground and the sole of the paw, thereby reducing the landing impact load (1).

By extracting the stress values of all nodes in the finite element model, the current research results found that the number of nodes with large stress values only accounts for 5–10% of the total number of nodes (Figure 4; Supplementary Table S6; Supplementary Figures S2–S12). In this case, it is often “fatal” to simply discuss the distribution of maximum stress values under different constraints just like in previous studies, because a large number of nodes without obvious changes may cover up the true nature of data change rules. As a result, the internal law of stress distribution feature cannot be truly excavated and cannot be effectively explored in terms the biomechanical characteristics of cat landing. Based on this, this study proposes for the first time to explore the inherent regularity of stress distribution characteristics by combining characteristic engineering techniques, to solve problems such as insufficient mining in the post-processing of FEA. By controlling the stress values corresponding to 5 different selected nodes as input data, the current study constructed the classification and recognition model to detect the recognition accuracy of the selected 12 stress distribution feature in different landing patterns at different heights. The results demonstrated that the classification and recognition are very low, only reaching about 50–60% (Figure 6; Supplementary Table S7), when the corresponding stress values of all nodes are taken as input data. As the input data of the model changes continuously (from all the stress values corresponding to the 3,300 nodes to the stress values corresponding to the first 2,000 nodes with the highest stress values, and finally to the stress values corresponding to the first 200 nodes with the highest stress values), the classification and recognition accuracy of each feature in landing patterns at different altitudes also continue to improve. In particular, for the metacarpal and proximal phalanges when the landing height is 0.8 and 1.0 m, the classification and recognition accuracies were almost 100% when the input data to the model was the stress values corresponding to the first 200 nodes with the highest stress values (Figure 6; Supplementary Table S7).

Regarding MP4 and PP2, the classification recognition accuracy rate of MP4 and PP2 was significantly higher in 5 different node selection cases than in other features. Meanwhile, both for the results based on the input data of landing from 0.8 to 1.0 m, and for the results based on the input data of landing from 1.0 to 1.2 m, among the features selected based on the feature selection model, there are many cases in which the stress distribution feature of the MP4 and PP2 were selected as the optimal feature to identify different landing patterns (Figure 5; Supplementary Table S6). This also suggests that MP4 and PP2 play an important role in cat landing. The results are different from those obtained by the traditional analysis method based on the comparison of stress distribution trend and maximum stress value. Traditional analysis results show that the stress distribution of cat landing is mainly concentrated in MP, especially in MP2, and its overall stress distribution is larger than that of other bones. In the feature selection model, the MP2 feature was selected as the optimal landing pattern recognition feature only when the data set *M*_*data*1_ was used as the model input data (*M*_*data*1_: based on the data of stress value corresponding to all nodes, when landing from 0.8 to 1.0 m). This suggests that a large number of nodes with no obvious change do indeed cover up the true nature of the data change law, so that the inherent law of stress distribution characteristics cannot be truly excavated. Therefore, considering only the maximum stress distribution does not effectively identify the features that contribute most to pattern recognition under different constraints, which is often not conducive to the grasp of the optimal feature that can reveal intrinsic properties in the field of biomechanics.

Another interesting result of the current work is that the stress distribution characteristics of the DP show low identifiability under almost all node selection conditions (Figure 6; Supplementary Table S7). At the same time, among the features selected based on the feature selection model, there are few cases in which the stress distribution feature of the DP is selected as the optimal feature to identify different landing patterns (Figure 5). This is also consistent with the results of the detailed stress distribution trend (Figure 4), that is, the stress distribution of the DP is much smaller than that of the MP and PP. This suggests that the DP may have played little role in cushioning the impact load when the cat landed. This may be due to the special physiological anatomical structure of the cat's paw pads fitting closely to the DP (36), which results in the cat's paw pads bearing most of the impact during the landing process, thereby reducing the force on the DP to avoid musculoskeletal injury. The recognition and classification accuracy of stress distribution features based on the input data of landing from 0.8 m and landing from 1.0 m is significantly higher than the results based on the input data of landing from 1.0 m and landing from 1.2 m (Figure 6; Supplementary Table S7). The results indicate that the difference in landing characteristics between cats landing from 1.0 to 0.8 m is higher than that between cats landing from 1.0 to 1.2 m. Previous studies have shown that landing height can alter the contribution ratio of skeletal energy dissipation in the forelimbs and hindlimbs, thereby reducing the risk of injury in cats landing from greater heights (9, 47). From the point of view of muscle activation, limb muscles become tense before initial ground contact, and the amount and timing of muscle activity are adjusted to avoid landing injuries (48–50). Therefore, when the landing height is increased from 0.8 to 1.0 m, the variations in landing pattern differences may be a response of the cat's forelimb by adjusting the musculoskeletal structure to reduce the risk of injury with a more optimal landing strategy.

There are some limitations inherent in the present study that need to be considered. The results of the current work are constrained by the breed, weight, sex, and age of the cats tested. Based on this, in our follow-up study, we intend to expand the diversity of the test sample to verify the reliability and applicability of the current findings. Another factor to consider is that the current study takes into account the economics and operability of modeling and simulation in the FEA, so it does not take into account the change of the specific position of the ligament during the landing. While this may have some minor impact on the results, it is acceptable when combined with the performability of the entire simulation.

## Conclusion

The current work investigated the cat forelimb paw biomechanical characteristics when landing from different heights by using the FEA, as well as first proposed to combine the feature engineering techniques for post-processing of FEA. The stress distribution feature of the MP2, MP4, PP2, and PP3 are the features that contribute most to landing pattern recognition when a cat landed under different constraints. The DP may have played little role in cushioning the impact load when the cat landed. With the landing altitude increases, the variations in landing pattern differences may be a response of the cat's forelimb by adjusting the musculoskeletal structure to reduce the risk of injury with a more optimal landing strategy. The combination of feature engineering techniques can effectively identify the features that contribute most to pattern recognition under different constraints, which is conducive to the grasp of the optimal feature that can reveal intrinsic properties in the field of biomechanics.

## Data availability statement

The original contributions presented in the study are included in the article/Supplementary material, further inquiries can be directed to the corresponding author.

## Ethics statement

The animal study was reviewed and approved by the Animal Care and Use Ethics Committee of Ningbo University. Written informed consent was obtained from the owners for the participation of their animals in this study.

## Author contributions

DX, HZ, and YG conceived the idea of the study. DX, HZ, QZ, JB, UU, and ZR collected the data. DX, HZ, QZ, XM, FG, and MW performed the analyses. DX, HZ, QZ, and YG drafted the manuscript. JB, UU, ZR, XM, FG, and YG provided major comments and revisions. All authors contributed critically to the drafts and gave final approval for publication.

## Funding

This study was sponsored by the Zhejiang Provincial Key Research and Development Program of China (2021C03130), Zhejiang Provincial Natural Science Foundation of China for Distinguished Young Scholars (LR22A020002), Philosophy and Social Sciences Project of Zhejiang Province, China (22QNYC10ZD and 22NDQN223YB), Educational Science Planning Project of Zhejiang Province (2021SCG083), the Fundamental Research Funds for the Provincial Universities of Zhejiang (SJWY2022014), Public Welfare Science and Technology Project of Ningbo, China (2021S134), Teaching Research Project of Ningbo University (JYXMXZD2022008 and JYXMXYB2021018), and K. C. Wong Magna Fund in Ningbo University.

## Conflict of interest

The authors declare that the research was conducted in the absence of any commercial or financial relationships that could be construed as a potential conflict of interest.

## Publisher's note

All claims expressed in this article are solely those of the authors and do not necessarily represent those of their affiliated organizations, or those of the publisher, the editors and the reviewers. Any product that may be evaluated in this article, or claim that may be made by its manufacturer, is not guaranteed or endorsed by the publisher.
