# Supplementary material for: A new method proposed to explore the feline's paw bones of contributing most to landing pattern recognition when landed under different constraints
Source: Front Vet Sci. 2022 Oct 10;9:1011357. doi: 10.3389/fvets.2022.1011357 (PMC9589501; doi:10.3389/fvets.2022.1011357)
Supplement: Supplementary file 1 [file Data_Sheet_1.PDF]

Supplementary Material

1 Supplementary Data

1.1 Supplementary Text 1 - Full details of materials and methods

Principal component analysis reconstructed data waveform of ground reaction force

In this study, Separate PCAs were conducted for each direction (X-axis: anterior and posterior GRF; Y-axis: lateral and medial GRF; Z-axis: vertical GRF) in each height (0.8 m, 1.0 m, 1.2 m) resulting in nine analyses. For each PCA, 20 sets of data were designed, and each set corresponded to 101 data points, combined into a 101×20 $(n \times p)$ matrix. The 20-dimensional vector constituted by these 20 groups of data is the original variable X:

$$X = \begin{bmatrix} x_{11} & x_{12} & \cdots & x_{1p} \\ x_{n1} & x_{22} & \cdots & x_{2p} \\ \vdots & \ddots & \ddots & \vdots \\ x_{n1} & x_{n2} & \cdots & x_{np} \end{bmatrix} = (x_1, x_2, \ldots, x_p)$$

Where n represents 101 data points after interpolation, and using $t(i)$ $(i = 1, 2, \ldots, n)$ to represent the specific time point during the landing phase. The cat had a certain landing position at each time point, which corresponded to a particular vector in the GRF. The matrix X was first normalized, and the covariance matrix $Cov(X)$ was calculated based on normalized matrix. Then, the eigenvalues λ_i and orthogonalized unit eigenvectors α_i were calculated from the $Cov(X)$. The eigenvalues λ_i obeying ranking $\lambda_1 \geq \lambda_2 \geq \cdots \geq \lambda_p \geq 0$ with $\sum_{i=1}^{p} \lambda_i = 1$. The orthogonalized unit eigenvectors a_i is the coefficient of PC scores PC_i with respect to the original variable X. The PC scores PC_i represent significant waveform characteristics, which contain the overall magnitude, timing differences and shape. The principal component $(PC_1, PC_2, \ldots, PC_m)$ to be selected is fixed by the accumulative contribution rate of variance information $G(m) = \frac{\sum_{i=1}^{m} \lambda_i}{\sum_{k=1}^{p} \lambda_k}$. The number of PC scores PC_i were determined based on the $G(m)$. Finally, the selected PC scores were multiplied by the transpose of the PC coefficient matrix, and each sample was multiplied by the sample's standard deviation vector plus the mean vector to reconstruct waveforms (principal GRF) [1, 2]. The GRF value in three directions at the time point of maximal elbow flexion were obtained from the reconstructed waveform then loaded into Mimics software to FEA.

Finite element analysis technology simulated the bone stress distribution of cat claw

The main bony, cartilage, and ligament components were automatically embedded into the solid soft tissue by FE model software. In the simulation calculation, the lower limb bones of the cat, ligaments, paw pad soft tissue and other materials were regarded as linear, isotropic, and uniform materials [1, 3, 4]. Specific material parameters were shown in **Supplementary Table 1** [1, 5-8]. In this study, the cat scratch pad was set as a hyperelastic material. In a previously published research paper, the biomechanics of the cat claw pad was studied, which was described as a nonlinear

viscoelastic property similar to that of a polymer [1, 9]. Therefore, a hyperelastic material model with quadratic polynomial strain energy potential was used to simulate the soft tissue wrapped by cat claws. Based on the uniaxial stress-strain data obtained from published studies, the soft tissue values of the claw were calculated by ANSYS Workbench17.0 under three loading frequencies (0.11Hz, 1.1Hz, 11Hz). This is because the behavior of hyperelastic materials was usually characterized by uniaxial test, biaxial test and shear test. The engineering stress and strain data were directly imported into the processor of ANSYS Workbench17.0.

The superior surface of the encapsulated solid part, distal tibia, and distal fibula was fixed. The paw pad-ground interaction was simulated as a paw pad-plate system, a commonly employed method during biomechanical modeling of the human foot. The plate was assigned with elastic property to simulate concrete ground support, and was allowed to move freely in all directions. The plate was assigned with elastic property to simulate concrete ground support, and was allowed to move freely in all directions. A vertical GRF was applied underneath the plate, which created a frictional contact ($\mu = 0.6$) with the paw. Other contacts were set to "bond". By changing the size of GRF in X, Y, Z-directions, the GRF landing from 0.8m, 1m and 1.2m was simulated respectively. The detailed GRF value for each landing height of the maximum elbow flexion was obtained based on the reconstructed waveform.

Optimal feature selection of landing patterns based on bone stress distribution

Data was entered five times in each of the two comparisons, including 1) the stress values corresponding to all the nodes (3300 nodes); 2) the stress values corresponding to the first 2000 nodes with the highest stress values; 3) the stress values corresponding to the first 1000 nodes with the highest stress values; 4) the stress values corresponding to the first 500 nodes with the highest stress values; 5) the stress values corresponding to the first 200 nodes with the highest stress values. Therefore, the feature selection algorithm model was run for a total of 10 times, and the data set for input model each time was as follows:

(1) $M_{data1} = 6600_{stress\ values}(3300_{stress\ values} \times 2_{landing\ from\ 0.8\ m\ and\ 1.0\ m}) \times 12_{bones}(4_{metacarpal} + 4_{proximal\ phalanx} + 4_{distal\ phalanx})$;

(2) $M_{data2} = 6600_{stress\ values}(3300_{stress\ values} \times 2_{landing\ from\ 1.0\ m\ and\ 1.2\ m}) \times 12_{bones}(4_{metacarpal} + 4_{proximal\ phalanx} + 4_{distal\ phalanx})$;

(3) $M_{data3} = 4000_{stress\ values}(2000_{stress\ values} \times 2_{landing\ from\ 0.8\ m\ and\ 1.0\ m}) \times 12_{bones}(4_{metacarpal} + 4_{proximal\ phalanx} + 4_{distal\ phalanx})$;

(4) $M_{data4} = 4000_{stress\ values}(2000_{stress\ values} \times 2_{landing\ from\ 1.0\ m\ and\ 1.2\ m}) \times 12_{bones}(4_{metacarpal} + 4_{proximal\ phalanx} + 4_{distal\ phalanx})$;

(5) $M_{data5} = 2000_{stress\ values}(1000_{stress\ values} \times 2_{landing\ from\ 0.8\ m\ and\ 1.0\ m}) \times 12_{bones}(4_{metacarpal} + 4_{proximal\ phalanx} + 4_{distal\ phalanx})$;

(6) $M_{data6} = 2000_{stress\ values}(1000_{stress\ values} \times 2_{landing\ from\ 1.0\ m\ and\ 1.2\ m}) \times 12_{bones}(4_{metacarpal} + 4_{proximal\ phalanx} + 4_{distal\ phalanx})$;

(7) $M_{data7} = 1000_{stress\ values}(500_{stress\ values} \times 2_{landing\ from\ 0.8\ m\ and\ 1.0\ m}) \times 12_{bones}(4_{metacarpal} + 4_{proximal\ phalanx} + 4_{distal\ phalanx})$;

(8) $M_{data8} = 1000_{stress\ values}(500_{stress\ values} \times 2_{landing\ from\ 1.0\ m\ and\ 1.2\ m}) \times 12_{bones}(4_{metacarpal} + 4_{proximal\ phalanx} + 4_{distal\ phalanx})$;

(9) $M_{data9} = 400_{stress\ values}(200_{stress\ values} \times 2_{landing\ from\ 0.8\ m\ and\ 1.0\ m}) \times 12_{bones}(4_{metacarpal} + 4_{proximal\ phalanx} + 4_{distal\ phalanx})$;

(10) $M_{data10} = 400_{stress\ values}(200_{stress\ values} \times 2_{landing\ from\ 1.0\ m\ and\ 1.2\ m}) \times 12_{bones}(4_{metacarpal} + 4_{proximal\ phalanx} + 4_{distal\ phalanx})$.

The algorithm implementation steps of BPSO as shown in **Supplementary Table 2**, in a Ddimensional issue, the V is the velocity of the particle:$V = (v_{i1}, v_{i2}, \dots, v_{iD})$. X is the position of the particle: $X = (x_{i1}, x_{i2}, \dots, x_{iD})$. where i is the particle's order in the population. In BPSO, each particle's best position is referred to as $Pbest$, and the population's global best solution is referred to as $Gbest$. To maintain the balance between global and local exploration, the inertia weight is progressively dropped from a higher value to a lower one. At each iteration, the inertia weight is calculated as:

$$w(t) = w_{max} - (w_{max} - w_{min})\frac{t}{T} \quad (1)$$

Where w_{max} and w_{min} are the inertia weight's bounds, t is the present iteration, and T is the maximum iteration numbers. The particle's velocity V is updated as follows for each iteration t:

$$v_i^d(t+1) = w(t) \times v_i^d(t) + c_1 \times r_1 \times \left(Pbest_i^d(t) - x_i^d(t)\right) + c_2 \times r_2 \times \left(Gbest^d(t) - x_i^d(t)\right) \quad (2)$$

Where x is the particle's position, v is the particle's velocity, i is the particle's order in the population, d is the search space's dimension, w is the inertia weight, c_1 and c_2 are the coefficients of acceleration. r_1 and r_2 both are independently generated random numbers, have a uniform distribution between 0 and 1. Then, using the sigmoid function, the velocity is transformed into a probability value as follow:

$$S(v_i^d(t+1)) = \frac{1}{1 + e^{-v_i^d(t+1)}} \quad (3)$$

Then, the particle's position is updated as:

$$x_i^d(t+1) = \begin{cases} 1, & If\ \delta \le S(v_i^d(t+1)) \\ 0, & Otherwise \end{cases} \quad (4)$$

Where δ is also a random number, that has a uniform distribution between 0 and 1.

The K-nearest neighbor algorithm (KNN) was first proposed by Cover and Hart in 1968, and it is mainly used in character recognition, text classification, image recognition and other fields [10]. The

algorithm idea of the KNN is that if a sample is most similar to k samples in the dataset, and most of the K samples belong to a certain category, then the sample also belongs to that category [10, 11]. Let the eigenspace χ be the n-dimensional vector space R^n of real numbers, $x_i, x_j \in \chi \subseteq R^n$, $i = 1,2,\dots,N$, $x_i = (x_i^{(1)}, x_i^{(2)}, \dots, x_i^{(n)})^T$, $x_j = (x_j^{(1)}, x_j^{(2)}, \dots, x_j^{(n)})^T$, and the L_p distance of x_i, x_j is defined as:

$$L_p(x_i, x_j) = \left(\sum_{l=1}^{n} |x_i^{(l)} - x_j^{(l)}|^p \right)^{\frac{1}{p}} \quad (5)$$

Where the p is a variable parameter, when $p = 2$ called the Euclidean distance, that is $L_2(x_i, x_j) = \left(\sum_{l=1}^{n} |x_i^{(l)} - x_j^{(l)}|^2 \right)^{\frac{1}{2}}$. In this study, the Euclidean distance $k = 5$, and the KNN was used since it is a fast, simple and common machine learning algorithm, which also has been widely used in feature selection research [10-13].

Feature classification and recognition based on bone stress distribution

For the KNN, the Euclidean distance k was set to 5. Support vector machine (SVM) was first proposed by Cortes and Vapnik in 1995, which has many unique advantages in solving nonlinear, small-sample, high-dimensional data pattern recognition problems [14]. Given training sample set $D = \{(x_1, y_1), (x_2, y_2), \dots, (x_m, y_m)\}, y_i \in \{-1, +1\}$, the essence of classification is to find a partition hyperplane in the sample space to separate samples of different categories. For the SVM, the model corresponding to the hyperplane partition in the feature space can be expressed as: $f(x) = \omega^T \phi(x) + b$. Where the $\omega = (\omega_1; \omega_2; \dots; \omega_d)$ is the normal vector that determines the direction of the hyperplane; the b is the displacement term, which determines the distance between the hyperplane and the origin; the $\phi(x)$ represents the eigenvectors after the x map. By setting the kernel function $\kappa(x_i, y_i) = \phi(x_i)^T \phi(x_j)$, the support vector expansion can be obtained:

$$f(x) = \omega^T \phi(x) + b = \sum_{i=1}^{m} \alpha_i y_i \phi(x_i)^T \phi(x) + b = \sum_{i=1}^{m} \alpha_i y_i \kappa(x, x_i) + b \quad (6)$$

In this study, the linear kernel functions ($\kappa(x, x_i) = {x_i}^T x_j$) were used to turn the input feature's data into a higher-dimensional space. At the same time, the soft margin idea was used to cope with the possibility of misclassifications. The soft margin should make the samples that do not meet the constraints as small as possible while maximizing the margin, then the optimization objective can be expressed as: $min_{\omega,b,\xi_i} \frac{1}{2}||\omega||^2 + C \sum_{i=1}^{m} \xi_i$. Where the ξ_i is the slack variables, and the C ($C = 1$) is a regularization constant [14, 15].

Artificial neural networks (ANN) are extensive parallel networks comprised of adaptable basic units whose organization can be used to replicate the interactions of organic nervous systems with real-world objects [16]. The linear relationship function of the model constructed in this study is $z = \sum_{i=1}^{m} w_i \, x_i + b$, where the w_i is the connection weight of the i-th neuron, the x_i is the input from the i-th neuron. The input layer, hidden layer, and output layer all were set to one in this study, and batch size was set as the 25, the max epoch was set to 1000 [1, 17]. Take the input x and run it linearly to

get z, and then the Sigmoid ($S = \frac{1}{1+e^{-x}}$) type activation function is used to get a. Finally, the neural network output a_i^{l+1} of the i-th neuron at layer $l + 1$ is:

$$f(x) = a_i^{l+1} = \sigma\left(z_i^{l+1}\right) = \sigma(\sum_i a_i^{(l)} w_{ij}^{(l,l+1)} + b_j^{(l+1)}) \quad (7)$$

The node of the input layer was determined according to the number of input features, the node of the hidden layer was determined according to the group number of input data, and the node of output layer was determined based on the number of class [1, 16, 18].

For the 10-fold cross-validation, the data were randomly divided into 10 parts, and then one of them was selected as the training set, and the remaining 9 parts were used as the test set, repeating a total of ten times.

1.2 Supplementary Text 2- Full details of results

Finite element model validation

The cat paw contact pressure and contact area distribution were also extracted from E-med measurement for comparison with simulated results (**Supplementary Figure 1**). During the verification process, the cat landed from a height of 1.2m, and the cat landed on the pressure platform providing measurement. The final ground reaction force (GRF) was 183.28N. For the validation of the cat's paw finite element (FE) model, the numerically predicted, and the experimentally obtained paw pressure distributions were compared. The paw pressure concentrated mainly on the metapodial pad with regard to the FE model or the experimental results. The numerically predicted contact area was approximately 58 cm2 in comparison to the experimentally obtained 55 cm2, which showed 5.4% higher over-prediction. The maximal pressure in the FE model was located at the metapodial pad in the measurement. The FE model predicted a peak pressure of 0.3667 MPa, while the experimental result, measured by the pressure platform, was 0.3 MPa, a difference of 22.2%.

Right forelimb paw stress distribution

Supplementary Figure 2 shows the detailed stress distribution heatmap and Pareto distribution results of stress values at all nodes of MP3, when landing at a height of 0.8 m, 1.0 m, and 1.2 m. For the MP3, the stress was mainly concentrated in the lower and front (**Supplementary Figure 2A,C,E**). The stress distribution ranges of all nodes were 0.0228-0.9830, 0.0299-0.3193, 0.0360-1.5196 respectively, the stress distribution ranges of the last 50% nodes were 0.0229-0.1956, 0.0299-0.2622, 0.0360-0.3035 respectively, the stress distribution ranges of the first 5% nodes were 0.6517-0.9830, 0.8741-0.3193, 1.0087-1.5196 respectively (**Supplementary Table 6, Supplementary Figure 2B,D,F**).

Supplementary Figure 3 shows the detailed stress distribution heatmap and Pareto distribution results of stress values at all nodes of MP4, when landing at a height of 0.8 m, 1.0 m, and 1.2 m. For the MP4, the stress was mainly concentrated in the middle and rear (**Supplementary Figure 3A,C,E**). The stress distribution ranges of all nodes were 0.0139-0.6125, 0.0299-0.8242, 0.0215-0.9449 respectively, the stress distribution ranges of the last 50% nodes were 0.0139-0.2285, 0.0183-0.3064, 0.0215-0.3542 respectively, the stress distribution ranges of the first 5% nodes were 0.5225-0.6125, 0.7022-0.8242, 0.8079-0.9449 respectively (**Supplementary Table 6, Supplementary Figure 3B,D,F**).

Supplementary Figure 4 shows the detailed stress distribution heatmap and Pareto distribution results of stress values at all nodes of MP5, when landing at a height of 0.8 m, 1.0 m, and 1.2 m. For the MP5, the stress was mainly concentrated in the middle and rear (**Supplementary Figure 4A,C,E**). The stress distribution ranges of all nodes were 0.0058-0.8713, 0.0079-1.1705, 0.0079-1.3462 respectively, the stress distribution ranges of the last 50% nodes were 0.0058-0.1884, 0.0079-0.2533, 0.0079-0.2910 respectively, the stress distribution ranges of the first 5% nodes were 0.6469-0.8713, 0.8694-1.1705, 1.0000-1.3462 respectively (**Supplementary Table 6, Supplementary Figure 4B,D,F**).

Supplementary Figure 5 shows the detailed stress distribution heatmap and Pareto distribution results of stress values at all nodes of PP2, when landing at a height of 0.8 m, 1.0 m, and 1.2 m. For the PP5, the stress was mainly concentrated in the middle (**Supplementary Figure 5A,C,E**). The stress distribution ranges of all nodes were 0.0066-0.2829, 0.0088-0.3806, 0.0102-0.4363 respectively, the stress distribution ranges of the last 50% nodes were 0.0066-0.1382, 0.0088-0.1858, 0.0102-0.2134 respectively, the stress distribution ranges of the first 5% nodes were 0.2304-0.2829, 0.3100-0.3806, 0.3559-0.4363 respectively (**Supplementary Table 6, Supplementary Figure 5B,D,F**).

Supplementary Figure 6 shows the detailed stress distribution heatmap and Pareto distribution results of stress values at all nodes of PP3, when landing at a height of 0.8 m, 1.0 m, and 1.2 m. For the PP3, the stress was mainly concentrated in the lower and rear (**Supplementary Figure 6A,C,E**). The stress distribution ranges of all nodes were 0.0102-0.2813, 0.0134-0.3748, 0.0163-0.4380 respectively, the stress distribution ranges of the last 50% nodes were 0.0102-0.1119, 0.0134-0.1485, 0.0163-0.1752 respectively, the stress distribution ranges of the first 5% nodes were 0.2004-0.2813, 0.2657-0.3748, 0.3141-0.4380 respectively (**Supplementary Table 6, Supplementary Figure 6B,D,F**).

Supplementary Figure 7 shows the detailed stress distribution heatmap and Pareto distribution results of stress values at all nodes of PP4, when landing at a height of 0.8 m, 1.0 m, and 1.2 m. For the PP4, the stress was mainly concentrated in the upper and front (**Supplementary Figure 7A,C,E**). The stress distribution ranges of all nodes were 0.0092-0.4172, 0.0118-0.5645, 0.0141-0.6395 respectively, the stress distribution ranges of the last 50% nodes were 0.0092-0.1270, 0.0118-0.1713, 0.0141-0.1956 respectively, the stress distribution ranges of the first 5% nodes were 0.3133-0.4172, 0.4240-0.5645, 0.4801-0.6395 respectively (**Supplementary Table 6, Supplementary Figure 7B,D,F**).

Supplementary Figure 8 shows the detailed stress distribution heatmap and Pareto distribution results of stress values at all nodes of PP5, when landing at a height of 0.8 m, 1.0 m, and 1.2 m. For the PP3, the stress was mainly concentrated in the upper and front (**Supplementary Figure 8A,C,E**). The stress distribution ranges of all nodes were 0.0310-0.4383, 0.0417-0.5887, 0.0479-0.6769 respectively, the stress distribution ranges of the last 50% nodes were 0.0310-0.1602, 0.0417-0.2153, 0.0479-0.2478 respectively, the stress distribution ranges of the first 5% nodes were 0.3187-0.4383, 0.4280-0.5887, 0.4925-0.6769 respectively (**Supplementary Table 6, Supplementary Figure 8B,D,F**).

Supplementary Figure 9 shows the detailed stress distribution heatmap and Pareto distribution results of stress values at all nodes of DP2, when landing at a height of 0.8 m, 1.0 m, and 1.2 m. For the DP2, the stress was mainly concentrated in the middle and front (**Supplementary Figure 9A,C,E**). The stress distribution ranges of all nodes were 0.0037-0.3000, 0.0052-0.4033, 0.0055-0.4634

respectively, the stress distribution ranges of the last 50% nodes were 0.0037-0.0392, 0.0052-0.0528, 0.0055-0.0606 respectively, the stress distribution ranges of the first 5% nodes were 0.1363-0.3000, 0.1831-0.4033, 0.2104-0.4634 respectively (**Supplementary Table 6, Supplementary Figure 9B,D,F**).

Supplementary Figure 10 shows the detailed stress distribution heatmap and Pareto distribution results of stress values at all nodes of DP3, when landing at a height of 0.8 m, 1.0 m, and 1.2 m. For the DP2, the stress was mainly concentrated in the middle and rear (**Supplementary Figure 10A,C,E**). The stress distribution ranges of all nodes were 0.0063-0.3269, 0.0086-0.4364, 0.0097-0.5085 respectively, the stress distribution ranges of the last 50% nodes were 0.0063-0.0662, 0.0086-0.0887, 0.0097-0.1030 respectively, the stress distribution ranges of the first 5% nodes were 0.2216-0.3269, 0.2950-0.4364, 0.3474-0.5085 respectively (**Supplementary Table 6, Supplementary Figure 10B,D,F**).

Supplementary Figure 11 shows the detailed stress distribution heatmap and Pareto distribution results of stress values at all nodes of DP4, when landing at a height of 0.8 m, 1.0 m, and 1.2 m. For the DP4, the stress was mainly concentrated in the middle and rear (**Supplementary Figure 11A,C,E**). The stress distribution ranges of all nodes were 0.0024-0.2381, 0.0033-0.3198, 0.0039-0.3677 respectively, the stress distribution ranges of the last 50% nodes were 0.0024-0.0408, 0.0033-0.0545, 0.0039-0.0634 respectively, the stress distribution ranges of the first 5% nodes were 0.1368-0.2381, 0.1829-0.3198, 0.2120-0.3677 respectively (**Supplementary Table 6, Supplementary Figure 11B,D,F**).

Supplementary Figure 12 shows the detailed stress distribution heatmap and Pareto distribution results of stress values at all nodes of DP5, when landing at a height of 0.8 m, 1.0 m, and 1.2 m. For the DP5, the stress was mainly concentrated in the upper and rear (**Supplementary Figure 12A,C,E**). The stress distribution ranges of all nodes were 0.0073-0.3181, 0.0099-0.4260, 0.0112-0.4926 respectively, the stress distribution ranges of the last 50% nodes were 0.0073-0.0733, 0.0099-0.0980, 0.0112-0.1136 respectively, the stress distribution ranges of the first 5% nodes were 0.1639-0.3181, 0.2197-0.4260, 0.2535-0.4926 respectively (**Supplementary Table 6, Supplementary Figure 12B,D,F**).

Feature selection results based on the bone stress distribution

For the data of landing from 0.8 m and landing from 1.0 m: 1) when the data of stress value corresponding to all nodes were substituted into the feature selection model as input data, the top three features that have been selected the most times were the PP5, PP3, MP2; 2) when the data of stress value corresponding to the first 2000 nodes with the highest stress values were substituted into the feature selection model as input data, the top three features that have been selected the most times were the PP5, PP2, MP3; 3) when the data of stress value corresponding to the first 1000 nodes with the highest stress values were substituted into the feature selection model as input data, the top three features that have been selected the most times were the PP3, MP3, PP2; 4) when the data of stress value corresponding to the first 500 nodes with the highest stress values were substituted into the feature selection model as input data, the top three features that have been selected the most times were the MP4, DP4, PP4; 5) when the data of stress value corresponding to the first 200 nodes with the highest stress values were substituted into the feature selection model as input data, the top three features that have been selected the most times were the PP2, MP4, DP3.

For the data of landing from 1.0 m and landing from 1.2 m: 1) when the data of stress value corresponding to all nodes were substituted into the feature selection model as input data, the top three features that have been selected the most times were the PP5, MP5, PP2; 2) when the data of stress value corresponding to the first 2000 nodes with the highest stress values were substituted into the feature selection model as input data, the top three features that have been selected the most times were the PP2, PP3, MP3; 3) when the data of stress value corresponding to the first 1000 nodes with the highest stress values were substituted into the feature selection model as input data, the top three features that have been selected the most times were the MP3, PP3, MP4; 4) when the data of stress value corresponding to the first 500 nodes with the highest stress values were substituted into the feature selection model as input data, the top three features that have been selected the most times were the DP2, MP4, DP4; 5) when the data of stress value corresponding to the first 200 nodes with the highest stress values were substituted into the feature selection model as input data, the top three features that have been selected the most times were the PP2, MP4, PP5.

Feature classification and recognition results based on the bone stress distribution

For the results based on the input data of landing from 0.8 m and landing from 1.0 m, the stress distribution features of PP2 show high recognizability than those of other bones in 5 different node selection cases (**Supplementary Figure 4A**, **Supplementary Table 7**). Specifically, for the results based on the data of the all nodes with the highest stress values, the stress distribution features of PP2 (62.50%), PP3 (60.92%), PP5 (62.33%) shows a high recognition accuracy. For the results based on the data of the first 2000 nodes with the highest stress values, the stress distribution features of PP2 (73.28%), PP3 (69.88%) shows a high recognition accuracy. For the results based on the data of the first 1000 nodes with the highest stress values, the stress distribution features of MP4 (79.12%), PP2 (87.22%), PP3 (80.17%) shows a high recognition accuracy. For the results based on the data of the first 500 nodes with the highest stress values, the stress distribution features of MP4 (97.17%), PP2 (94.33%), PP4 (95.83%) shows a high recognition accuracy. For the results based on the data of the first 200 nodes with the highest stress values, the stress distribution features of MP2 (92.58%), MP4 (100.00%), MP5 (96.42%), PP2 (100.00%), PP4 (99.00%), PP5 (96.50%) shows a high recognition accuracy.

For the results based on the data of landing from 1.0 m and landing from 1.2 m, in the case based on the data of the all nodes with the highest stress values, the stress distribution features of PP2 (54.72%), PP3 (56.27%), PP5 (57.07%) shows a high recognition accuracy. For the results based on the data of the first 2000 nodes with the highest stress values, the stress distribution features of PP3 (61.62%) shows a high recognition accuracy. For the results based on the data of the first 1000 nodes with the highest stress values, the stress distribution features of PP2 (67.02%), PP3 (69.35%) shows a high recognition accuracy. For the results based on the data of the first 500 nodes with the highest stress values, the stress distribution features of MP4 (73.27%), PP2 (73.03%), PP3 (73.33%) shows a high recognition accuracy. For the results based on the data of the first 200 nodes with the highest stress values, the stress distribution features of MP4 (81.25%) shows a high recognition accuracy.

2 Supplementary Figures and Tables

2.1 Supplementary Figures

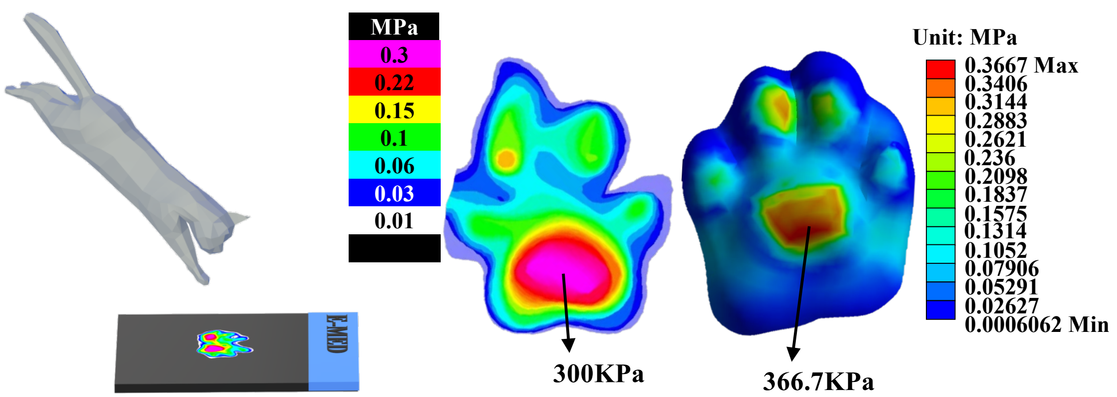

Supplementary Figure 1. Illustration of the process of model validation, the stress distribution of experiment and finite element simulation on the paw pad of cat.

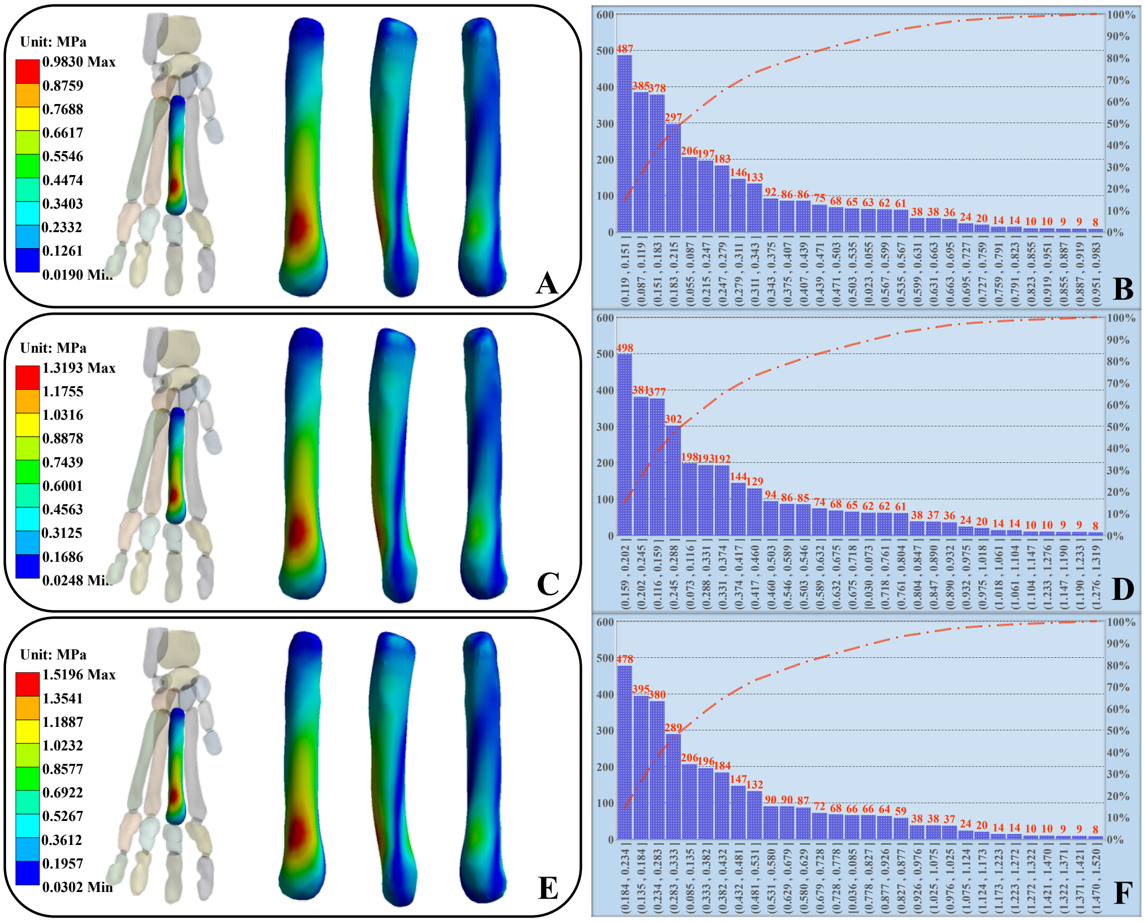

Supplementary Figure 2. The detailed stress distribution heatmap (A, C, E) and Pareto distribution (B, D, F) results of stress values at all nodes of MP3. The stress distribution from top to bottom

corresponds to the landing heights of 0.8 m, 1.0 m and 1.2 m. From left to right are front view, side view and back view of the MP3.

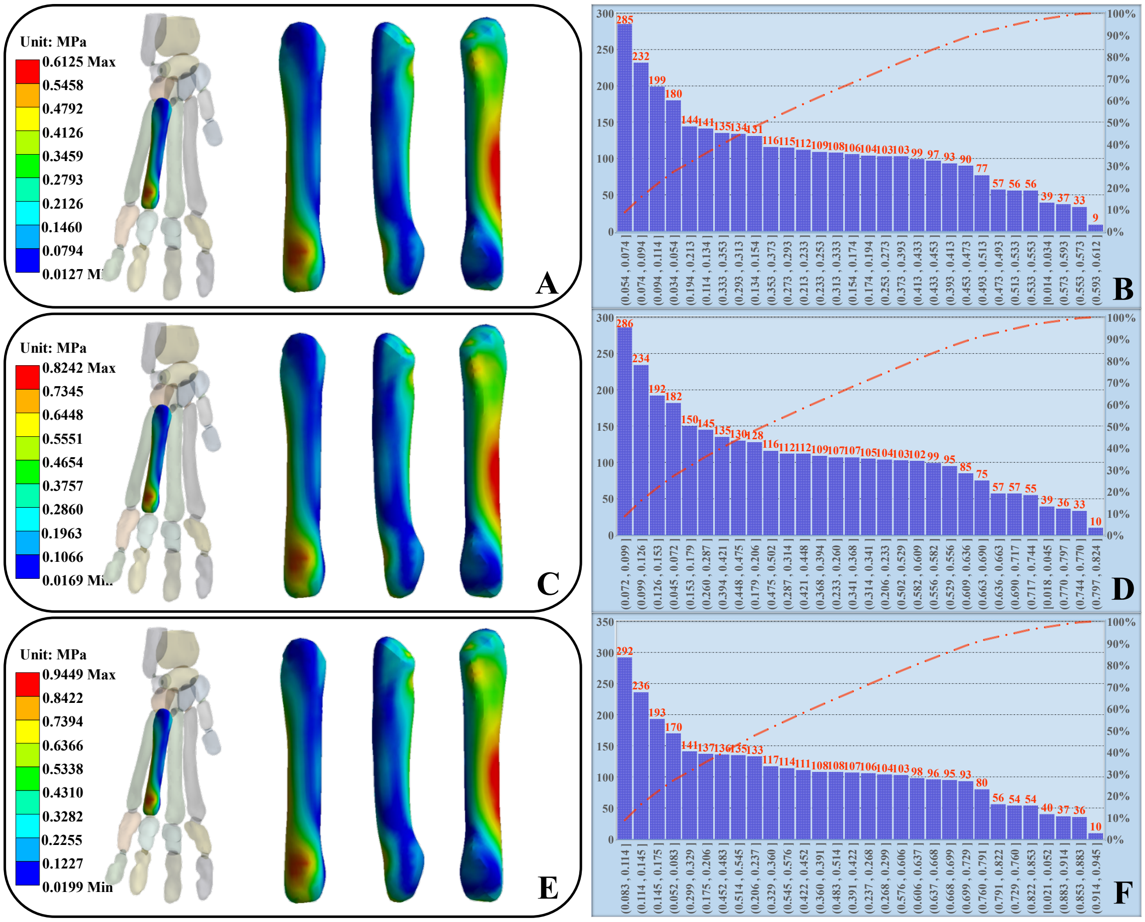

Supplementary Figure 3. The detailed stress distribution heatmap (A, C, E) and Pareto distribution (B, D, F) results of stress values at all nodes of MP4. The stress distribution from top to bottom corresponds to the landing heights of 0.8 m, 1.0 m and 1.2 m. From left to right are front view, side view and back view of the MP4.

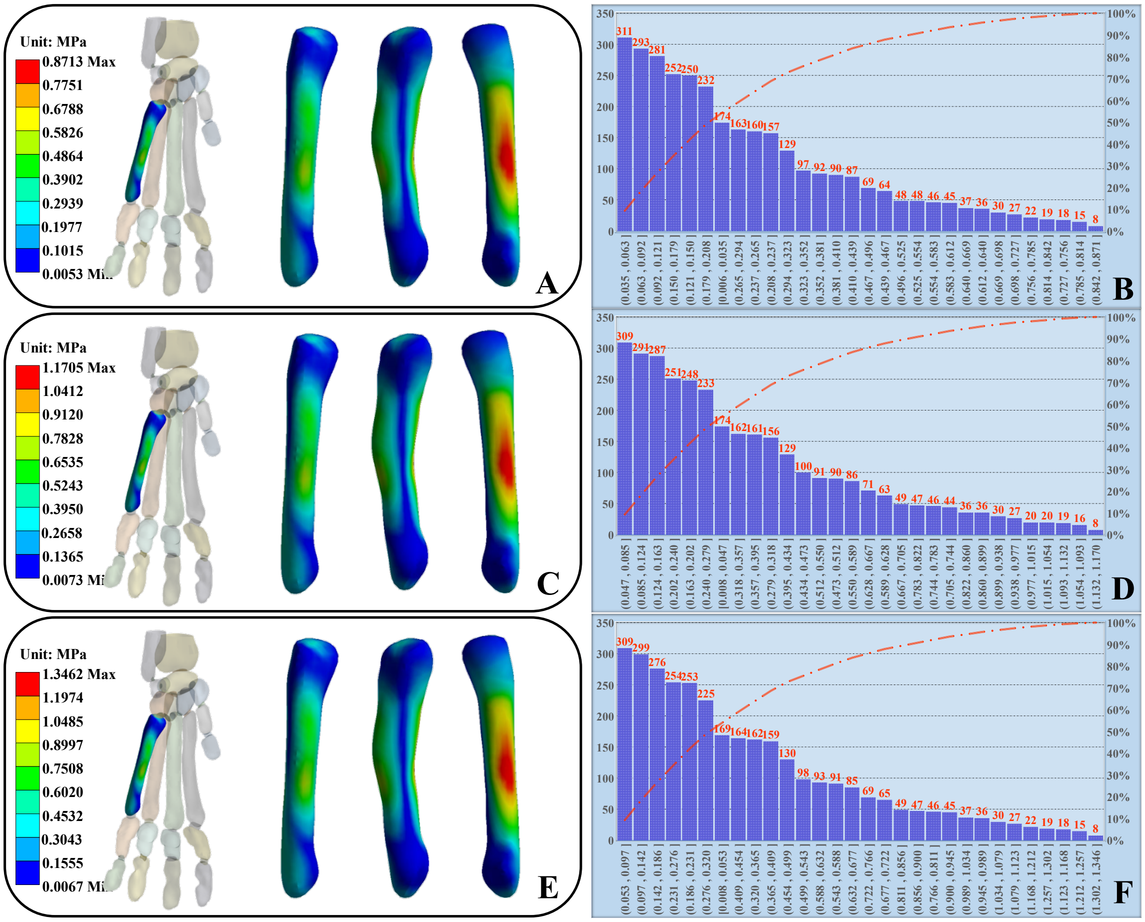

Supplementary Figure 4. The detailed stress distribution heatmap (A, C, E) and Pareto distribution (B, D, F) results of stress values at all nodes of MP5. The stress distribution from top to bottom corresponds to the landing heights of 0.8 m, 1.0 m and 1.2 m. From left to right are front view, side view and back view of the MP5.

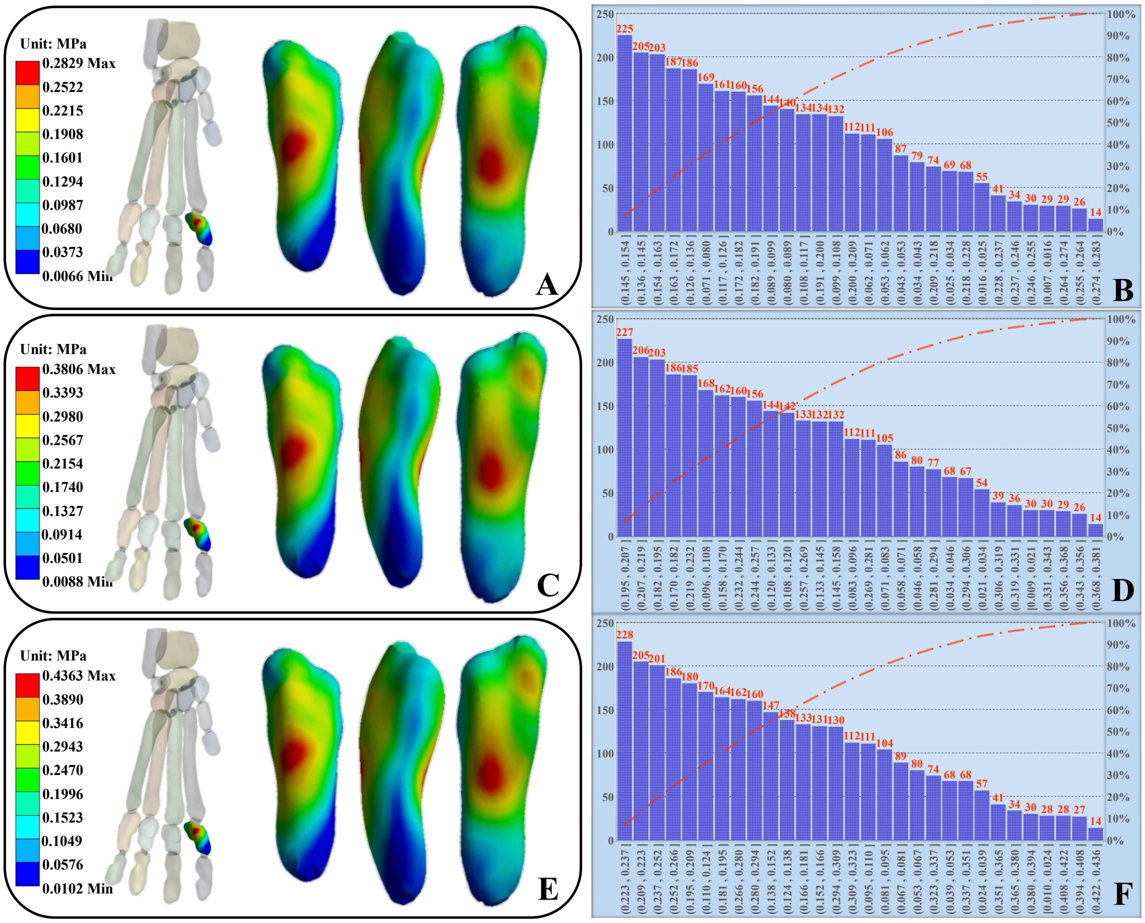

Supplementary Figure 5. The detailed stress distribution heatmap (A, C, E) and Pareto distribution (B, D, F) results of stress values at all nodes of PP2. The stress distribution from top to bottom corresponds to the landing heights of 0.8 m, 1.0 m and 1.2 m. From left to right are front view, side view and back view of the PP2.

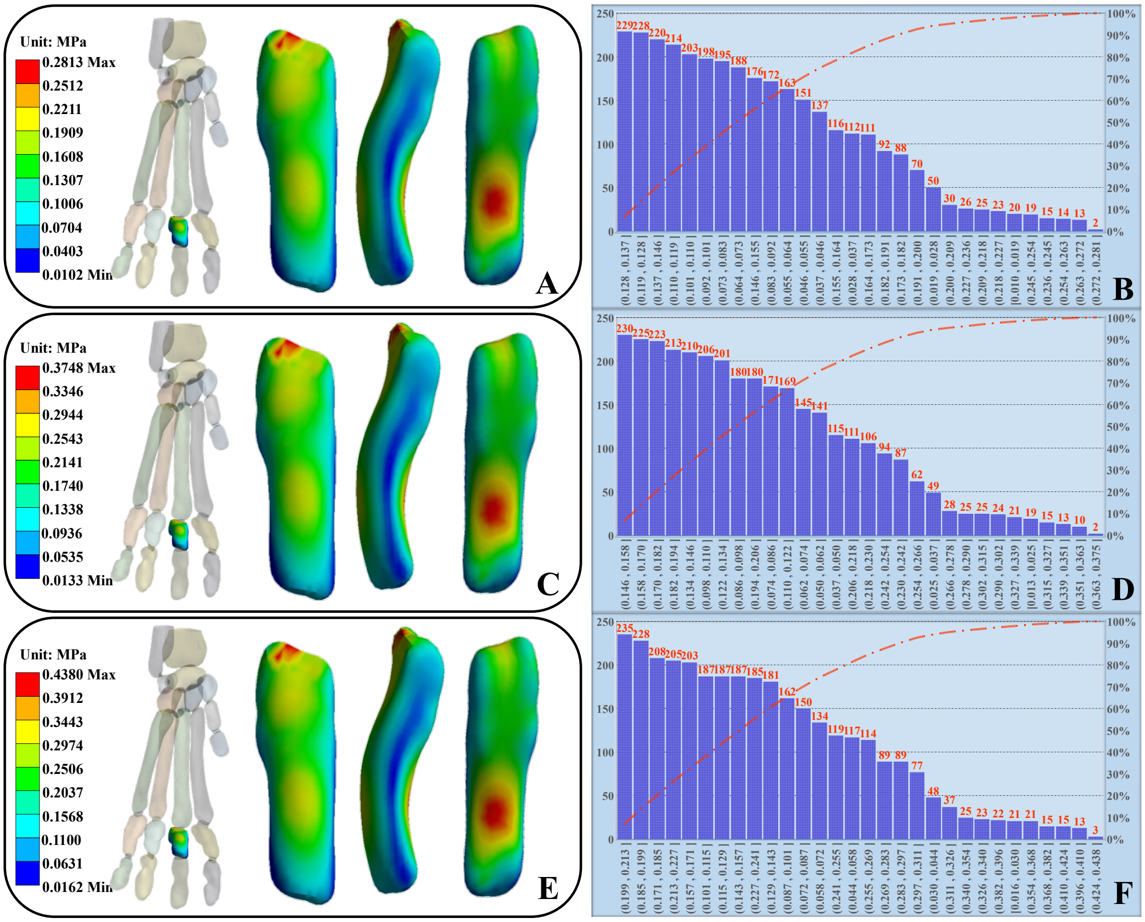

Supplementary Figure 6. The detailed stress distribution heatmap (A, C, E) and Pareto distribution (B, D, F) results of stress values at all nodes of PP3. The stress distribution from top to bottom corresponds to the landing heights of 0.8 m, 1.0 m and 1.2 m. From left to right are front view, side view and back view of the PP3.

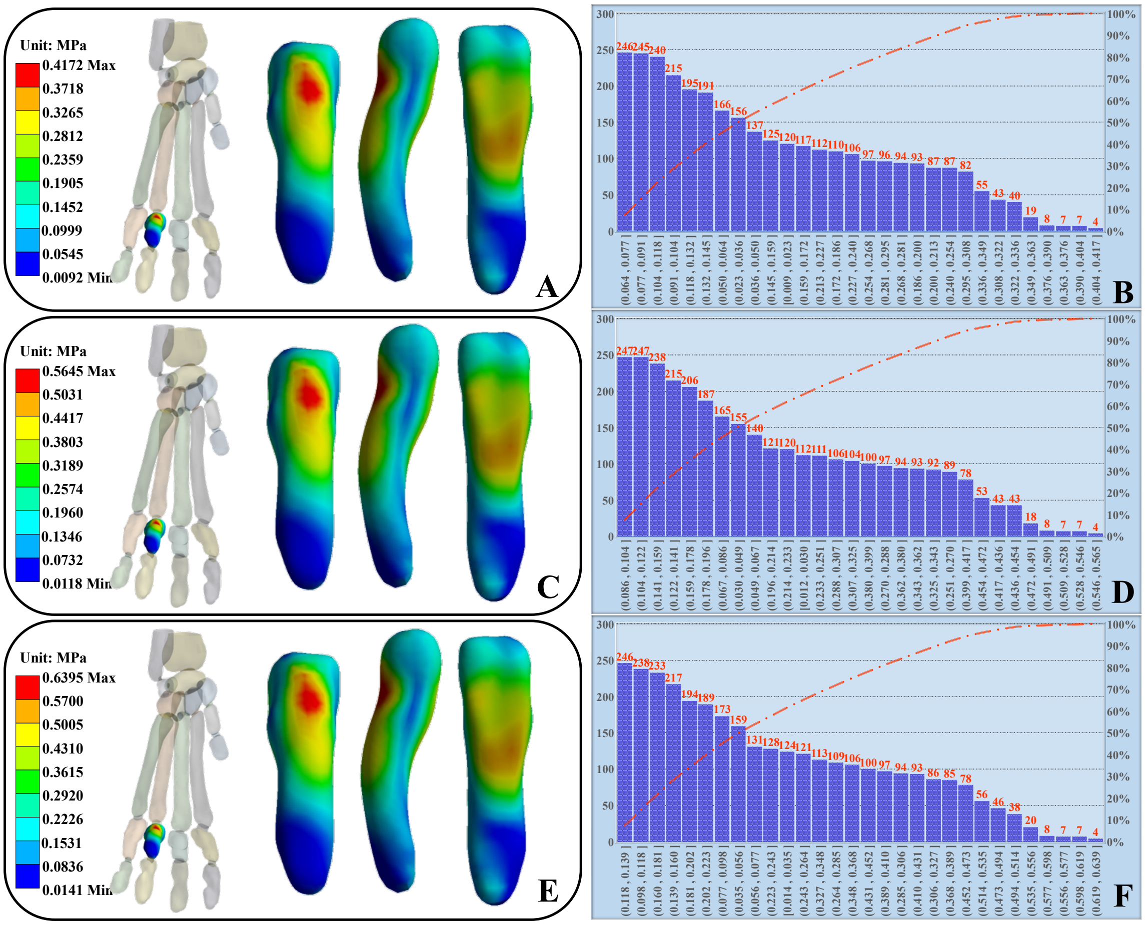

Supplementary Figure 7. The detailed stress distribution heatmap (A, C, E) and Pareto distribution (B, D, F) results of stress values at all nodes of PP4. The stress distribution from top to bottom corresponds to the landing heights of 0.8 m, 1.0 m and 1.2 m. From left to right are front view, side view and back view of the PP4.

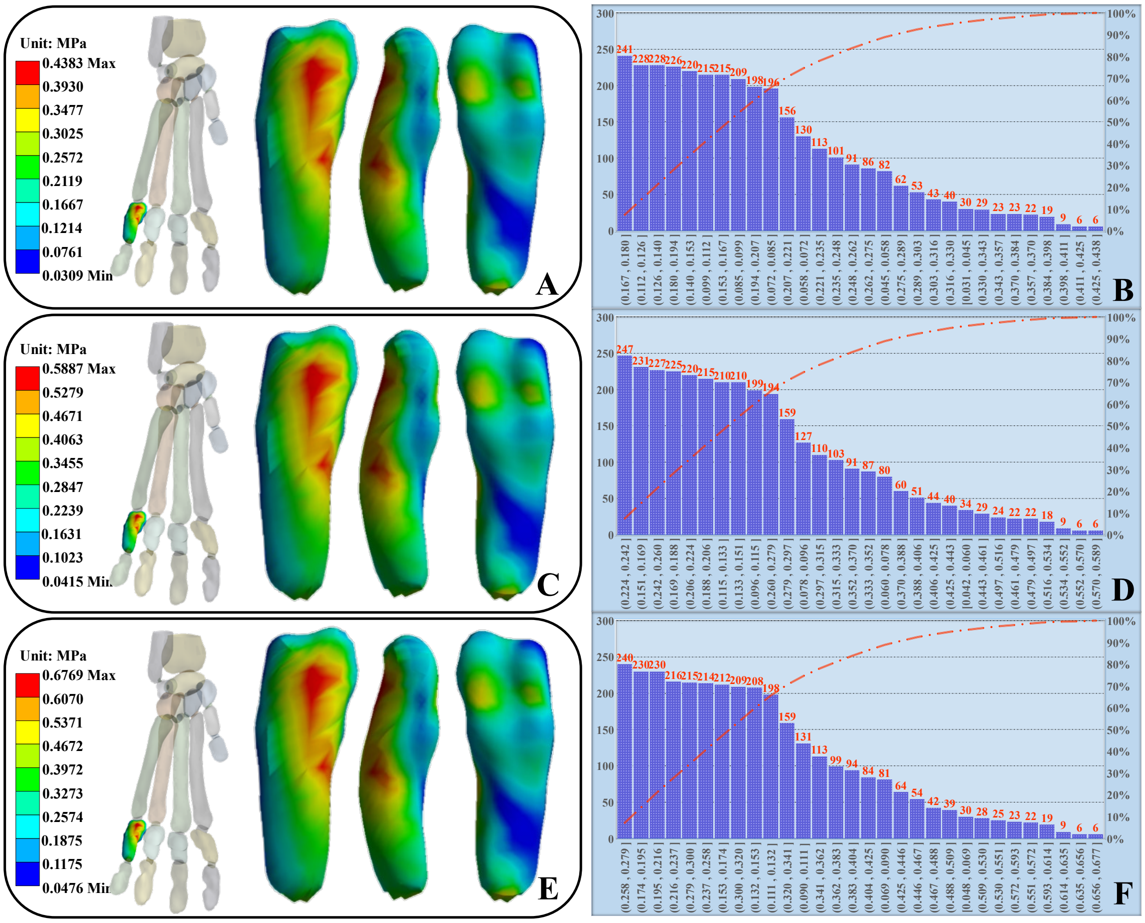

Supplementary Figure 8. The detailed stress distribution heatmap (A, C, E) and Pareto distribution (B, D, F) results of stress values at all nodes of PP5. The stress distribution from top to bottom corresponds to the landing heights of 0.8 m, 1.0 m and 1.2 m. From left to right are front view, side view and back view of the PP5.

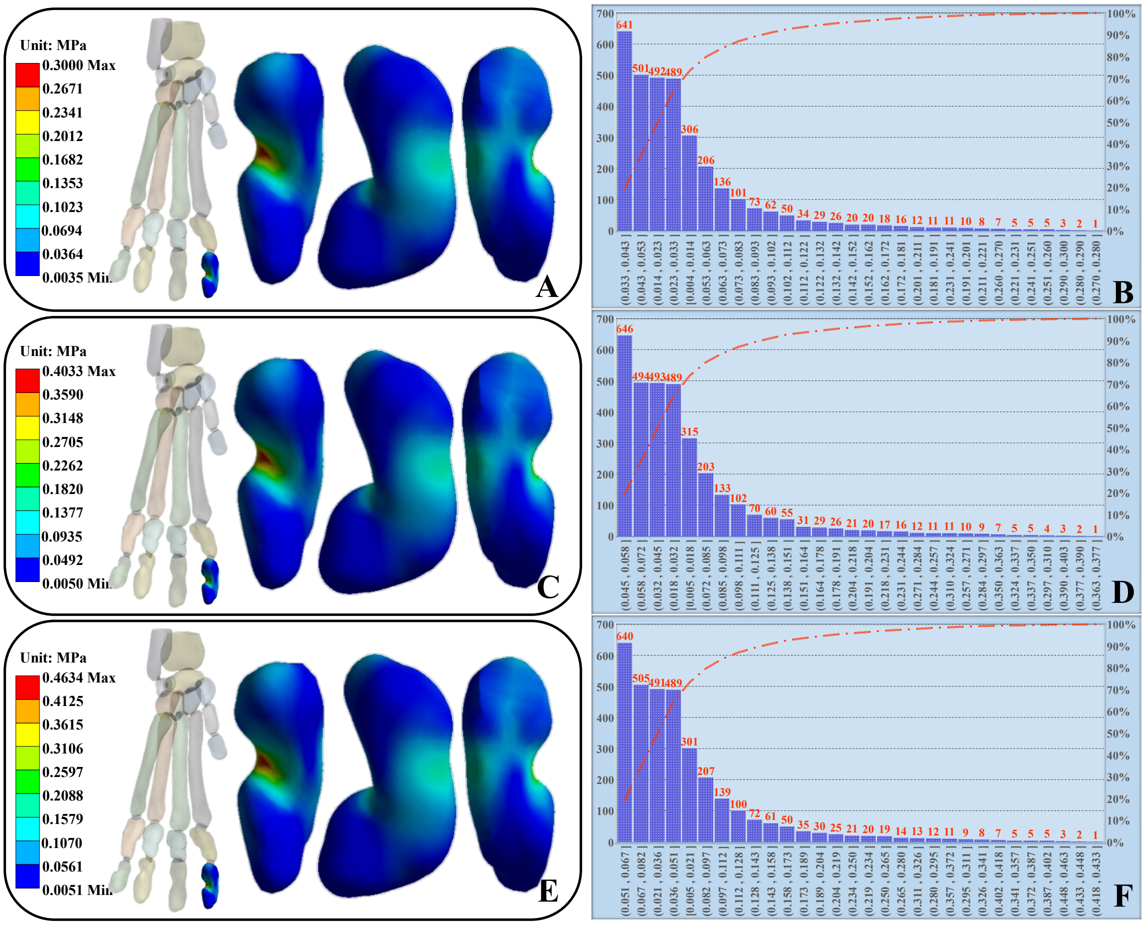

Supplementary Figure 9. The detailed stress distribution heatmap (A, C, E) and Pareto distribution (B, D, F) results of stress values at all nodes of DP2. The stress distribution from top to bottom corresponds to the landing heights of 0.8 m, 1.0 m and 1.2 m. From left to right are front view, side view and back view of the DP2.

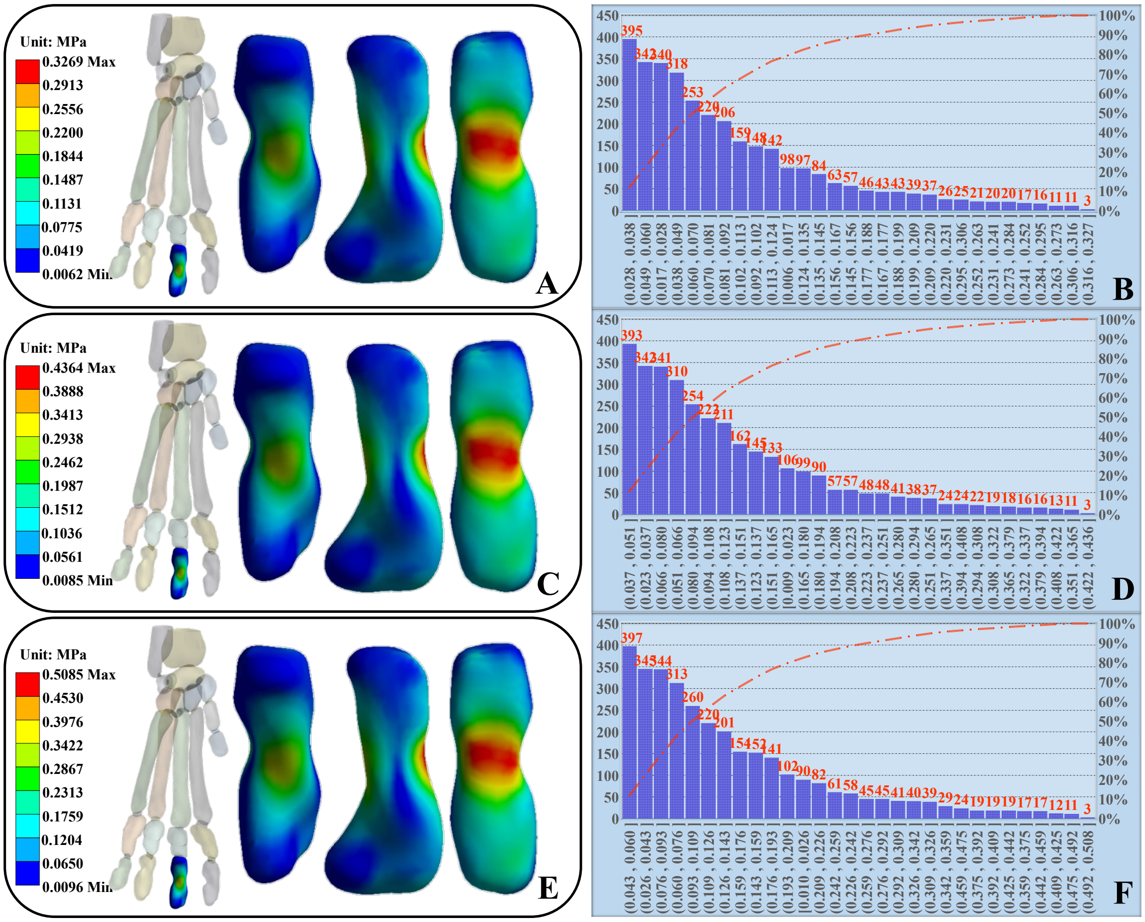

Supplementary Figure 10. The detailed stress distribution heatmap (A, C, E) and Pareto distribution (B, D, F) results of stress values at all nodes of DP3. The stress distribution from top to bottom corresponds to the landing heights of 0.8 m, 1.0 m and 1.2 m. From left to right are front view, side view and back view of the DP3.

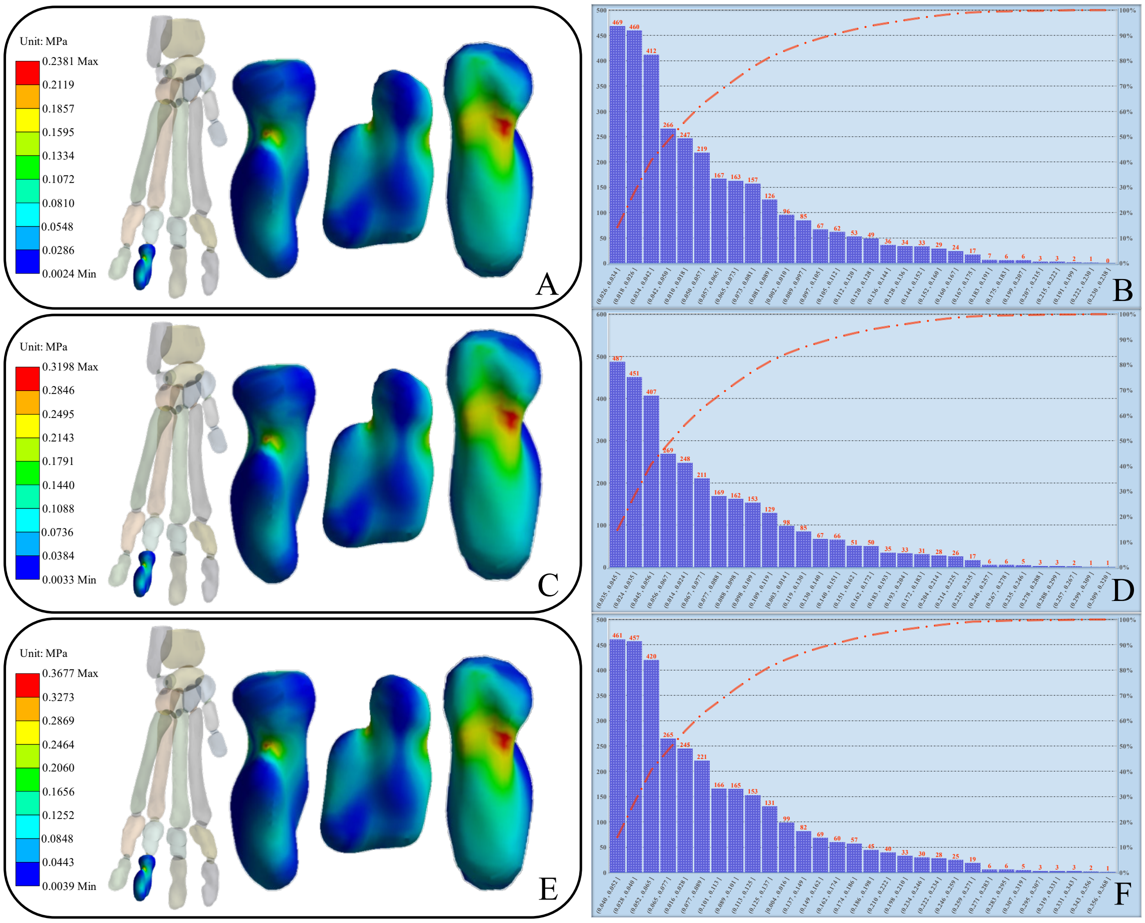

Supplementary Figure 11. The detailed stress distribution heatmap (A, C, E) and Pareto distribution (B, D, F) results of stress values at all nodes of DP4. The stress distribution from top to bottom corresponds to the landing heights of 0.8 m, 1.0 m and 1.2 m. From left to right are front view, side view and back view of the DP4.

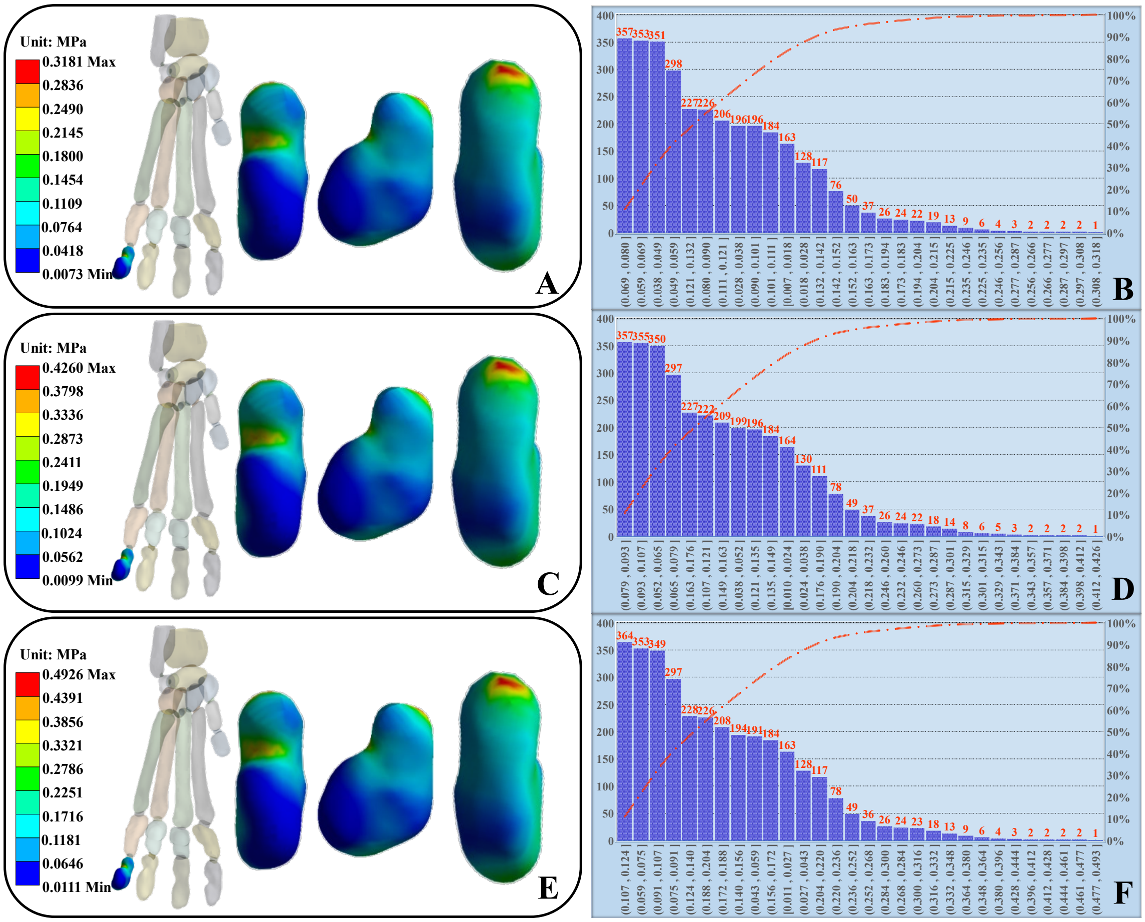

Supplementary Figure 12. The detailed stress distribution heatmap (A, C, E) and Pareto distribution (B, D, F) results of stress values at all nodes of DP5. The stress distribution from top to bottom corresponds to the landing heights of 0.8 m, 1.0 m and 1.2 m. From left to right are front view, side view and back view of the DP5.

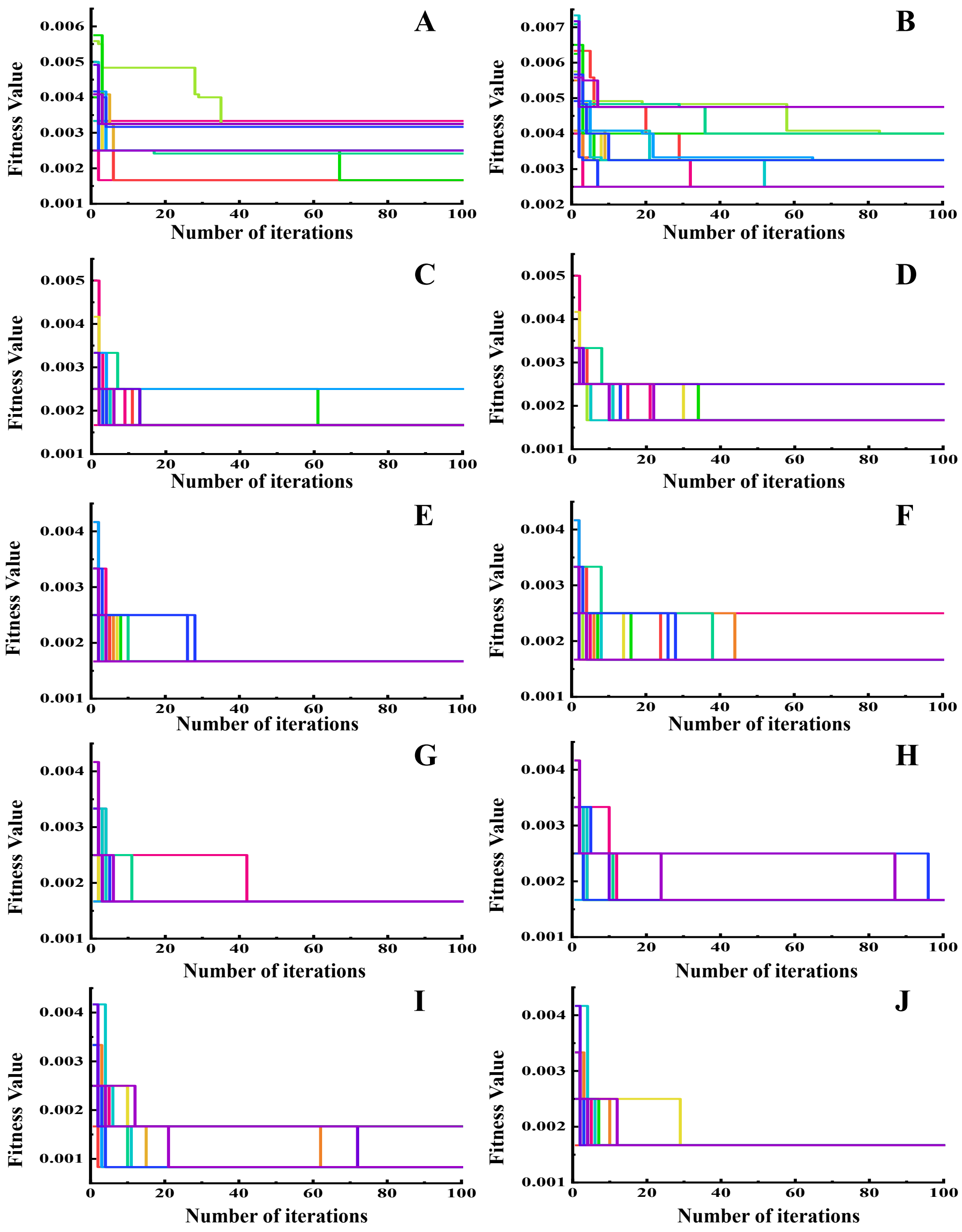

Supplementary Figure 13. Fitness value curve of 20 random seeds in 10 different contrasting situations. For the data of landing from 0.8 m and landing from 1.0 m, (A) (C) (E) (G) (I) are the results based on the data of stress value corresponding to all nodes, the data of the first 2000 nodes with the highest stress values, the data of the first 1000 nodes with the highest stress values, the data of the first 500 nodes with the highest stress values, the data of the first 200 nodes with the highest stress values, respectively. For the data of landing from 1.0 m and landing from 1.2 m, (B) (D) (F) (H) (J) are the results based on the data of stress value corresponding to all nodes, the data of the first 2000 nodes with the highest stress values, the data of the first 1000 nodes with the highest stress values, the data of the first 500 nodes with the highest stress values, the data of the first 200 nodes with the highest stress values, respectively.

2.2 Supplementary Tables

Supplementary Table 1. The material parameters of cat paw pad model components.

Component	Young's Modulus E (MPa)	Poisson's Ratio ν
Paw	0.15	0.45
Bone	15000	0.3
Cartilage	1	0.4
Ligaments	260	0.4
Plate	17000	0.4

Supplementary Table 2. The algorithm implementation steps of BPSO.

Binary Particle Swarm Optimization
$Begin;$
$Initialize\ the\ parameters:\ N,\ T_{max},\ c_1, c_2,\ v_{max},\ v_{min};$
$Innitialize\ the\ parrticles's\ population:\ X;$
$Evaluate\ fitness\ of\ particles:\ F(X);$
$Set\ pbest,\ gbest;$
$for\ t = 1; Maximum\ number\ of\ iterations:\ T_{max};$
$Calculate\ the\ interia\ weight\ w\ (Eq.\ 1);$
$for\ i = 1,\ the\ particles's\ number\ D;$
$for\ d = 1,\ the\ dimension's\ number\ D;$
$Update\ the\ particle's\ velocity\ v_{id}^{t+1}\ (Eq.\ 2);$
$Transform\ the\ velocity\ into\ probability\ values\ (Eq.\ 3);$
$Update\ the\ particle's\ position\ X_{id}^{t+1}\ (Eq.\ 4);$

$next\ d;$

$Evaluate\ fitness\ of\ new\ particle\ F(X_i^{t+1});$

$next\ i;$

$Update\ the\ pbest\ and\ gbest;$

$next\ t;$

$End;$

Supplementary Table 3. Comparison of forelimb raw in maximum force, peak and mean pressure and contact areas during landing from different heights.

Parameters	Landing heights	FL (Mean±SD)	FR (Mean±SD)	P value	MD±SED (95% CI)
Maximum Force (N)	0.8 m	176.02±8.99	172.20±8.53	0.176	3.82±2.77 (-1.79 to 9.43)
	1.0 m	199.71±8.89	198.55±9.38	0.690	1.16±2.89 (-4.69 to 7.01)
	1.2 m	230.68±21.00	225.15±18.34	0.381	5.53±6.23 (-7.09 to 18.15)
Peak Pressure (N/cm^2)	0.8 m	327.25±35.45	310.75±30.06	0.121	16.50±10.39 (-4.54 to 37.54)
	1.0 m	378.00±30.01	375.25±25.16	0.755	2.75±8.76 (-14.98 to 20.48)
	1.2 m	472.75±34.16	475.00±66.75	0.722	7.75±21.63 (-36.03 to 51.53)

Mean Pressure (N/cm^2)	0.8 m	110.10±5.24	110.05±6.29	0.978	0.05±1.83 (-3.65 to 3.75)
	1.0 m	122.95±5.79	120.47±4.79	0.149	2.47±1.68 (-0.93 to 5.88)
	1.2 m	141.54±11.89	135.3±11.88	0.110	6.15±3.76 (-1.46 to 13.76)
Contact Areas (cm^2)	0.8 m	9.58±0.55	9.86±0.75	0.186	-0.28±0.21 (-0.70 to 0.14)
	1.0 m	9.51±0.50	9.56±0.49	0.773	-0.05±0.16 (-0.36 to 0.27)
	1.2 m	10.94±1.04	10.73±0.87	0.484	0.22±0.30 (-0.40 to 0.83)

Note: FL: Forelimb paw left; FR: Forelimb paw right; M: Mean; SD: Standard deviation; MD: Mean difference; SED: Standard error deviation; CI: Confidence intervals. $p<0.05$ was set as a significant difference.

Supplementary Table 4. Comparison of forelimb paw right in maximum force, peak and mean pressure and contact areas during landing from different heights.

Parameters	0.8 m (Mean±SD)	1.0 m (Mean±SD)	1.2 m (Mean±SD)
Maximum Force (N)	176.02±8.99 ^{a,b}	199.71±8.89 ^{a,c}	230.68±21.00 ^{b,c}
Peak Pressure (N/cm^2)	327.25±35.45 ^{a,b}	378.00±30.01 ^{a,c}	482.75±69.99 ^{b,c}
Mean Pressure (N/cm^2)	110.10±5.2 ^{a,b}	122.95±5.79 ^{a,c}	141.54±11.89 ^{b,c}
Contact Areas (cm^2)	9.58±0.55 ^{b}	9.51±0.50 ^{c}	10.94±1.04 ^{b,c}

Note: a: There were significant differences between 0.8 m and 1.0 m; b: There were significant differences between 0.8 m and 1.2 m; c: There were significant differences between 1.0 m and 1.2 m. $p<0.05$ was set a significant difference.

Supplementary Table 5. The bone corresponding to each selected feature and its named abbreviation, as well as the number of nodes corresponding to its finite element model.

The number of Feature	Bones	Abbreviations	Number of nodes in finite element model
1	The second metacarpal	MP2	3309
2	The third metacarpal	MP3	3208
3	The fourth metacarpal	MP4	3217
4	The fifth metacarpal	MP5	3209
5	The second proximal phalanx	PP2	3499
6	The third proximal phalanx	PP3	3519
7	The fourth proximal phalanx	PP4	3565
8	The fifth proximal phalanx	PP5	3553
9	The second distal phalanx	DP2	3694
10	The third distal phalanx	DP3	3488
11	The fourth distal phalanx	DP4	3548
12	The fifth distal phalanx	DP5	3583

Supplementary Table 6. Each bones stress distribution ranges of all nodes and of the last 50%, 80%, 90%, 95% nodes, the first 5% nodes, when landing at a height of 0.8 m, 1.0 m, and 1.2 m.

Bones	Landing height	The last 50% nodes	The last 80% nodes	The last 90% nodes	The last 95% nodes	The first 5% nodes	All nodes
MP2	0.8 m	[0.0135, 0.2550]	[0.0135, 0.5480]	[0.0135, 0.7325]	[0.0135, 0.8772]	[0.8772, 1.2166]	[0.0135, 1.2166]
	1.0 m	[0.0179, 0.3418]	[0.0179, 0.7334]	[0.0179, 0.9794]	[0.0179, 1.1748]	[1.1748, 1.6274]	[0.0179, 1.6274]
	1.2 m	[0.0211, 0.3936]	[0.0211, 0.8497]	[0.0211, 1.1358]	[0.0211, 1.3588]	[1.3588, 1.8862]	[0.0211, 1.8862]
MP3	0.8 m	[0.0229, 0.1956]	[0.0229, 0.4024]	[0.0229, 0.5462]	[0.0229, 0.6517]	[0.6517, 0.9830]	[0.0228, 0.9830]
	1.0 m	[0.0299, 0.2622]	[0.0299, 0.5391]	[0.0299, 0.7302]	[0.0299, 0.8741]	[0.8741, 0.3193]	[0.0299, 0.3193]
	1.2 m	[0.0360, 0.3035]	[0.0360, 0.6230]	[0.0360, 0.8459]	[0.0360, 1.0087]	[1.0087, 1.5196]	[0.0360, 1.5196]
MP4	0.8 m	[0.0139, 0.2285]	[0.0139, 0.4005]	[0.0139, 0.4717]	[0.0139, 0.5225]	[0.5225, 0.6125]	[0.0139, 0.6125]

	1.0 m	[0.0183, 0.3064]	[0.0299, 0.5380]	[0.0299, 0.6340]	[0.0299, 0.7022]	[0.7022, 0.8242]	[0.0299, 0.8242]
	1.2 m	[0.0215, 0.3542]	[0.0215, 0.6191]	[0.0215, 0.7286]	[0.0215, 0.8079]	[0.8079, 0.9449]	[0.0215, 0.9449]
	0.8 m	[0.0058, 0.1884]	[0.0058, 0.3984]	[0.0058, 0.5351]	[0.0058, 0.6469]	[0.6469, 0.8713]	[0.0058, 0.8713]
MP5	1.0 m	[0.0079, 0.2533]	[0.0079, 0.5359]	[0.0079, 0.7194]	[0.0079, 0.8694]	[0.8694, 1.1705]	[0.0079, 1.1705]
	1.2 m	[0.0079, 0.2910]	[0.0079, 0.6145]	[0.0079, 0.8261]	[0.0079, 1.0000]	[1.0000, 1.3462]	[0.0079, 1.3462]
	0.8 m	[0.0066, 0.1382]	[0.0066, 0.1849]	[0.0066, 0.1849]	[0.0066, 0.2304]	[0.2304, 0.2829]	[0.0066, 0.2829]
PP2	1.0 m	[0.0088, 0.1858]	[0.0088, 0.2489]	[0.0088, 0.2794]	[0.0088, 0.3100]	[0.3100, 0.3806]	[0.0088, 0.3806]
	1.2 m	[0.0102, 0.2134]	[0.0102, 0.2851]	[0.0102, 0.3202]	[0.0102, 0.3559]	[0.3559, 0.4363]	[0.0102, 0.4363]
PP3	0.8 m	[0.0102, 0.1119]	[0.0102, 0.1539]	[0.0102, 0.1818]	[0.0102, 0.2004]	[0.2004, 0.2813]	[0.0102, 0.2813]

	1.0 m	[0.0134, 0.1485]	[0.0134, 0.2037]	[0.0134, 0.2405]	[0.0134, 0.2657]	[0.2657, 0.3748]	[0.0134, 0.3748]
	1.2 m	[0.0163, 0.1752]	[0.0163, 0.2415]	[0.0163, 0.2856]	[0.0163, 0.3141]	[0.3141, 0.4380]	[0.0163, 0.4380]
	0.8 m	[0.0092, 0.1270]	[0.0092, 0.2364]	[0.0092, 0.2861]	[0.0092, 0.3133]	[0.3133, 0.4172]	[0.0092, 0.4172]
PP4	1.0 m	[0.0118, 0.1713]	[0.0118, 0.3198]	[0.0118, 0.3861]	[0.0118, 0.4240]	[0.4240, 0.5645]	[0.0118, 0.5645]
	1.2 m	[0.0141, 0.1956]	[0.0141, 0.3637]	[0.0141, 0.4395]	[0.0141, 0.4801]	[0.4801, 0.6395]	[0.0141, 0.6395]
	0.8 m	[0.0310, 0.1602]	[0.0310, 0.2293]	[0.0310, 0.2761]	[0.0310, 0.3187]	[0.3187, 0.4383]	[0.0310, 0.4383]
PP5	1.0 m	[0.0417, 0.2153]	[0.0417, 0.3078]	[0.0417, 0.3708]	[0.0417, 0.4280]	[0.4280, 0.5887]	[0.0417, 0.5887]
	1.2 m	[0.0479, 0.2478]	[0.0479, 0.3538]	[0.0479, 0.4263]	[0.0479, 0.4925]	[0.4925, 0.6769]	[0.0479, 0.6769]
DP2	0.8 m	[0.0037, 0.0392]	[0.0037, 0.0632]	[0.0037, 0.0962]	[0.0037, 0.1363]	[0.1363, 0.3000]	[0.0037, 0.3000]

	1.0 m	[0.0052, 0.0528]	[0.0052, 0.0848]	[0.0052, 0.1294]	[0.0052, 0.1831]	[0.1831, 0.4033]	[0.0052, 0.4033]
	1.2 m	[0.0055, 0.0606]	[0.0055, 0.0976]	[0.0055, 0.1483]	[0.0055, 0.2104]	[0.2104, 0.4634]	[0.0055, 0.4634]
	0.8 m	[0.0063, 0.0662]	[0.0063, 0.1263]	[0.0063, 0.1778]	[0.0063, 0.2216]	[0.2216, 0.3269]	[0.0063, 0.3269]
DP3	1.0 m	[0.0086, 0.0887]	[0.0086, 0.1688]	[0.0086, 0.2368]	[0.0086, 0.2950]	[0.2950, 0.4364]	[0.0086, 0.4364]
	1.2 m	[0.0097, 0.1030]	[0.0097, 0.1964]	[0.0097, 0.2769]	[0.0097, 0.3474]	[0.3474, 0.5085]	[0.0097, 0.5085]
	0.8 m	[0.0024, 0.0408]	[0.0024, 0.0806]	[0.0024, 0.1091]	[0.0024, 0.1368]	[0.1368, 0.2381]	[0.0024, 0.2381]
DP4	1.0 m	[0.0033, 0.0545]	[0.0033, 0.1079]	[0.0033, 0.1461]	[0.0033, 0.1829]	[0.1829, 0.3198]	[0.0033, 0.3198]
	1.2 m	[0.0039, 0.0634]	[0.0039, 0.1243]	[0.0039, 0.1686]	[0.0039, 0.2120]	[0.2120, 0.3677]	[0.0039, 0.3677]
DP5	0.8 m	[0.0073, 0.0733]	[0.0073, 0.1206]	[0.0073, 0.1386]	[0.0073, 0.1639]	[0.1639, 0.3181]	[0.0073, 0.3181]

1.0 m	[0.0099, 0.0980]	[0.0099, 0.1614]	[0.0099, 0.1856]	[0.0099, 0.2197]	[0.2197, 0.4260]	[0.0099, 0.4260]
1.2 m	[0.0112, 0.1136]	[0.0112, 0.1866]	[0.0112, 0.2145]	[0.0112, 0.2535]	[0.2535, 0.4926]	[0.0112, 0.4926]

Supplementary Table 7. Total exact classification recognition accuracy rate obtained by the three classification models in each contrasting situation.

Landing height	The number of nodes	MP2	MP3	MP4	MP5	PP2	PP3	PP4	PP5	DP2	DP3	DP4	DP5
0.8 m VS 1.0 m	3300 nodes	54.5± 2.63	56.49± 2.54	57.68± 1.96	54.93± 2.33	62.50± 3.39	60.92± 3.45	57.18± 3.39	62.33± 2.05	57.02± 4.07	55.59± 2.42	56.15± 2.90	58.83± 2.80
	2000 nodes	58.42± 2.35	60.68± 3.47	63.79± 2.45	59.50± 2.09	73.28± 2.53	69.88± 2.74	64.30± 3.51	70.15± 2.50	62.87± 7.08	59.08± 2.88	59.93± 2.86	67.01± 2.97
	1000 nodes	66.42± 3.92	65.35± 2.89	79.12± 3.23	65.82± 2.71	87.22± 2.66	80.17± 3.83	76.03± 3.72	77.68± 3.94	62.43± 6.68	65.47± 4.92	66.75± 4.99	77.65± 5.81
	500 nodes	77.20± 4.30	78.00± 4.49	97.17± 1.56	76.77± 4.78	94.33± 2.25	84.73± 5.42	95.83± 1.52	87.83± 6.61	63.33± 4.97	69.13± 4.97	71.83± 5.27	77.47± 9.25

	200 nodes	92.58± 4.65	87.58± 5.69	100.00 ±0.00	96.42± 3.24	100.00 ±0.00	90.50± 6.32	99.00± 1.71	96.50± 2.46	69.67± 7.41	83.50± 7.00	80.58± 15.08	79.83± 10.18
1.0 m VS 1.2 m	3300 nodes	51.70± 2.38	53.07± 2.07	53.13± 2.17	52.04± 1.98	54.72± 3.56	56.27± 3.45	52.65± 2.17	57.07± 2.00	52.29± 3.08	52.25± 1.67	52.08± 3.09	54.02± 2.90
	2000 nodes	53.88± 2.28	55.59± 2.99	56.48± 2.33	54.85± 2.76	59.73± 3.25	61.62± 3.05	55.83± 2.47	59.91± 3.23	55.05± 4.15	54.20± 1.92	54.84± 2.78	59.64± 3.35
	1000 nodes	57.45± 4.97	58.08± 3.14	63.32± 3.43	57.43± 2.93	67.02± 4.81	69.35± 4.49	61.42± 3.59	65.33± 4.20	55.12± 4.39	59.23± 4.72	57.83± 4.03	61.95± 5.83
	500 nodes	63.23± 5.26	62.53± 4.70	73.27± 4.35	63.00± 4.90	73.03± 6.61	73.33± 8.29	70.80± 4.85	69.10± 5.23	55.97± 4.32	61.63± 5.77	59.97± 4.57	66.00± 8.38
	200 nodes	71.92± 8.30	68.67± 7.54	93.50± 4.03	73.17± 5.98	80.92± 6.58	74.67± 7.00	81.25± 9.21	76.50± 6.97	57.75± 6.48	71.50± 6.81	65.50± 10.39	63.25± 8.39

References:

1. Xu D, Zhou H, Jiang X, Li S, Zhang Q, Baker JS, et al. New Insights for the Design of Bionic Robots: Adaptive Motion Adjustment Strategies During Feline Landings. *Front Vet Sci*. (2022) 9:836043. doi: 10.3389/fbioe.2021.679123
2. Daffertshofer A, Lamoth CJ, Meijer OG, Beek PJ. PCA in studying coordination and variability: a tutorial. *Clin Biomech*. (2004) 19:415-428. doi: 10.1016/j.clinbiomech.2004.01.005
3. Böhme B, d'Otreppe V, Ponthot J-P, Balligand M. Intraosseous stress distribution and bone interaction during load application across the canine elbow joint: a preliminary finite element analysis for determination of condylar fracture pathogenesis in immature and mature dogs. *Res Vet Sci* (2016) 106:143-148. doi: 10.1016/j.rvsc.2016.03.014
4. Szabó B, Babuška I. Finite Element Analysis: Method, Verification and Validation. (2021). doi: 10.1002/9781119426479
5. BONSER RC. The Young's modulus of ostrich claw keratin. *J Mater Sci*. (2000) 19:1039-1040. doi: 10.1023/A:1006786919376
6. Zhou H, Li H, Mei Y, Wang G, Liu C, Zhang L. Research on vibration reduction method of nonpneumatic tire spoke based on the mechanical properties of domestic cat's paw pads. *Appl Bionics Biomech*. (2021) 2021. doi: 10.1155/2021/9976488
7. Kim SE, Arzi B, Garcia TC, Verstraete FJ. Bite forces and their measurement in dogs and cats. *Front Vet Sci*. (2018) 5:76. doi: 10.3389/fvets.2018.00076
8. Wang M, Song Y, Baker JS, Fekete G, Ugbolue UC, Li S, et al. The biomechanical characteristics of a feline distal forelimb: a finite element analysis study. *Comput Biol Med*. (2021) 129:104174. doi: 10.1016/j.compbiomed.2020.104174
9. Wu X, Pei B, Pei Y, Hao Y, Zhou K, Wang W. Comprehensive biomechanism of impact resistance in the cat's paw pad. *BioMed Res Int*. (2019) 2019. doi: 10.1155/2019/2183712
10. Cover T, Hart P. Nearest neighbor pattern classification. IEEE transactions on information theory. (1967) 13:21-27. doi: 10.1109/TIT.1967.1053964
11. Peterson LE. K-nearest neighbor. *Scholarpedia.* (2009) 4:1883. doi: 10.4249/scholarpedia.1883
12. Too J, Abdullah AR, Mohd Saad N. Hybrid binary particle swarm optimization differential evolution-based feature selection for EMG signals classification. *Axioms.* (2019) 8:79. doi: 10.3390/axioms8030079
13. Lu Z, Sun D, Xu D, Li X, Baker JS, Gu Y. Gait Characteristics and Fatigue Profiles When Standing on Surfaces with Different Hardness: Gait Analysis and Machine Learning Algorithms. *Biology.* (2021) 10:1083. doi: 10.3390-biology10111083
14. Cortes C, Vapnik V. Support-vector networks. *Mach Learn*. (1995) 20:273-297. doi: 10.1007/BF00994018
15. Noble WS. What is a support vector machine? *Nat Biotechnol*. (2006) 24:1565-1567. doi: 10.1038/nbt1206-1565
16. Kohonen T. An introduction to neural computing. *Neural Networks.* (1988) 1:3-16. doi: 10.1016/0893-6080(88)90020-2

17. Wang S-C. Artificial neural network. Interdisciplinary computing in java programming: Springer; 2003. p. 81-100. doi: 10.1007/978-1-4615-0377-4_5

18. Xu D, Quan W, Zhou H, Sun D, Baker JS, Gu Y. Explaining the differences of gait patterns between high and low-mileage runners with machine learning. *Sci Rep*. (2022) 12:1-12. doi: 10.1038/s41598-022-07054-1
